# Years of life lost due to traumatic brain injury in Europe: A cross-sectional analysis of 16 countries

**DOI:** 10.1371/journal.pmed.1002331

**Published:** 2017-07-11

**Authors:** Marek Majdan, Dominika Plancikova, Andrew Maas, Suzanne Polinder, Valery Feigin, Alice Theadom, Martin Rusnak, Alexandra Brazinova, Juanita Haagsma

**Affiliations:** 1 Department of Public Health, Faculty of Health Sciences and Social Work, Trnava University, Trnava, Slovakia; 2 Department of Neurosurgery, Antwerp University Hospital and University of Antwerp, Edegem, Belgium; 3 Department of Public Health, Erasmus University Medical Center, Rotterdam, The Netherlands; 4 National Institute for Stroke and Applied Neuroscience, Auckland University of Technology, Auckland, New Zealand; 5 Department of Emergency Medicine, Erasmus University Medical Center, Rotterdam, The Netherlands; Oregon Health and Science University, UNITED STATES

## Abstract

**Introduction:**

Traumatic brain injuries (TBIs) are a major public health, medical, and societal challenge globally. They present a substantial burden to victims, their families, and the society as a whole. Although indicators such as incidence or death rates provide insight into the occurrence and outcome of TBIs in various populations, they fail to quantify the full extent of their public health and societal impact. Measures such as years of life lost (YLLs), which quantifies the number of years of life lost because the person dies prematurely due to a disease or injury, should be employed to better quantify the population impact. The aim of this study was to provide an in-depth analysis of the burden of deaths due to TBI by calculating TBI-specific YLLs in 16 European countries, analyzing their main causes and demographic patterns, using data extracted from death certificates under unified guidelines and collected in a standardized manner.

**Methods and findings:**

A population-wide, cross-sectional epidemiological study was conducted in 16 European countries to estimate TBI YLLs for the year 2013. The data used for all analyses in this study were acquired from the statistical office of the European Union (Eurostat). A specifically tailored dataset of micro-level data was provided that listed the external cause of death (International Classification of Diseases–10th Revision [ICD-10] codes V01–Y98), the specific nature of injury (ICD-10 codes S00–T98), the age at death, and sex for each death. Overall number of TBI YLLs, crude and age-standardized TBI YLL rates, and TBI YLLs per case were calculated stratified for country, sex, and age. Pooled analyses were performed in order to estimate summary age-standardized rates of TBI YLLs. In order to evaluate the relative importance of TBI in the context of all injuries, proportions of TBI YLLs out of overall injury YLLs were calculated. The total number of TBI YLLs was estimated by extrapolating the pooled crude rate of TBI YLLs in the 16 analyzed countries to the total population of the 28 member states of the EU (EU-28). We found that a total of 17,049 TBI deaths occurred in 2013 in the 16 analyzed countries. These translated into a total of 374,636 YLLs. The pooled age-standardized rate of YLLs per 100,000 people per year was 259.1 (95% CI: 205.8 to 312.3) overall, 427.5 (95% CI: 290.0 to 564.9) in males, and 105.4 (95% CI: 89.1 to 121.6) in females. Males contributed substantially more to TBI YLLs than females (282,870 YLLs, 76% of all TBI YLLs), which translated into a rate ratio of 3.24 (95% CI: 3.22 to 3.27). Each TBI death was on average associated with 24.3 (95% CI: 22.0 to 26.6) YLLs overall, 25.6 (95% CI: 23.4 to 27.8) in males and 20.9 (17.9 to 24.0) in females. Falls and traffic crashes were the most common external causes of TBI YLLs. TBI contributed on average 41% (44% in males and 34% in females) to overall injury YLLs. Extrapolating our findings, about 1.3 million YLLs were attributable to TBI in the EU-28 in 2013 overall, 1.1 million in males and 271,000 in females. This study is based on administratively collected data from 16 countries, and despite the efforts to harmonize them to the greatest possible extent, there may be differences in coding practices or reporting between countries. If present, these would be inherited into our findings without our ability to control for them. The extrapolation of the pooled rates from the 16 countries to the EU-28 should be interpreted with caution.

**Conclusions:**

Our study showed that TBI-related deaths and YLLs have a substantial impact at the individual and population level in Europe and present an important societal and economic burden that must not be overlooked. We provide information valuable for policy-makers, enabling them to evaluate and plan preventive activities and resource allocation, and to formulate and implement strategic decisions. In addition, our results can serve as a basis for analyzing the overall burden of TBI in the population.

## Abstract 2

Marek Majdan and colleagues use detailed data from the statistical office of the European Union to estimate the burden of deaths due to traumatic brain injury in 16 European countries.

## Author summary

**Why was this study done?:**

- Traumatic brain injuries (TBIs) are an important health and societal problem—every year 57,000 TBI-related deaths and 1.5 million TBI-related hospital admissions occur in the European union.
- Preventive action and policies aimed to improve the situation must rely on detailed and valid epidemiological data on the occurrence and impact of TBI.
- Measures of population health such as years of life lost (YLLs) have been designed to capture the consequences of deaths due to specific illnesses in the population in more detail than traditional epidemiological indicators, such as mortality rates.

**What did the researchers do and find?:**

- We used YLLs to evaluate the burden of TBI deaths using detailed data from 16 European countries.
- We found that, in the analyzed countries, a total of 374,636 years of life were lost due to deaths caused by TBI, with the majority (76%) occurring in the male population, which translated to an summary rate of 259.1 TBI YLLs per 100,000 persons per year in the EU.
- Each TBI death was on average associated with 24.3 YLLs.
- Our findings extrapolated to the whole population of the EU suggest that about 1.3 million YLLs were attributable to TBI in the EU overall in 2013, with 1.1 million in males and 270,000 in females.

**What do these findings mean?:**

- Our findings are important for policy-makers to plan specifically tailored preventive actions to avoid TBI-related deaths, and thus reduce the number of YLLs that are associated with them.
- The results of our study confirm TBI-related deaths as an important public health concern, and they can serve as the basis for further research on this topic.
- To our knowledge, this is to date the largest and most comprehensive study of TBI YLLs in Europe.

## Introduction

Traumatic brain injuries (TBIs) are a major public health, medical, and societal challenge globally [1–4]. In the European Union alone, an estimated 57,000 deaths and 1.5 million hospital admissions annually have been attributed to TBI. This translates to a pooled population mortality rate of 11.7 (95% CI: 9.9 to 13.6) and hospital admission rate of 287.2 (95% CI: 232.9 to 341.5) per 100,000 persons per year [5]. The overall mortality in persons following TBI has been shown to be substantially higher than mortality in the general population—a pooled standardized mortality ratio of 2.18 (95% CI: 1.88–2.52) [6]. Life expectancy after TBI has been estimated to range from less than 40% to over 85% of that of the general population—depending on the severity of the injury and the level of impairment [7]. Due to the long-term character of the disabilities after TBI and their unstable nature (e.g., deterioration of previously achieved levels of outcome occurs in about 1 of 3 patients within 10 years post-injury), TBI has been considered a chronic condition [8]. Thus, the general burden of TBI to victims, their families, and the society as a whole is substantial and has been well documented.

Recent epidemiological research suggests that the patterns of TBI are dynamic, that they are changing over time, and that they are dependent on the demographic structure of the population and the level of economic development [9]. In emerging economies, intensive motorization that is not accompanied by adequate and enforced preventive measures has led to a substantial increase in TBIs related to traffic crashes [2,9]. As life expectancy has increased in high-income countries, TBI from falls has become more prevalent [9–11]. In order to cope with such variation, and to achieve any improvements in the levels of occurrence and outcomes of TBI, standardized, reproducible, regularly updated, and comparable epidemiological data are needed [5,12,13]. Most published epidemiological studies on TBI have focused on using case fatality rates, population mortality, or incidence to describe the epidemiology of TBI [14–16].

Although these indicators provide insight into the occurrence and outcome of TBIs in various populations, they fail to quantify the full extent of their public health and societal impact. Summary measures of population health used in the Global Burden of Disease Study have been designed to capture mortality and morbidity impact, and to allow subsequent comparison of disease impact on public health across a range of illnesses and populations. Among these measures are years of life lost (YLLs), which quantifies the number of years of life lost because the person dies prematurely due to a disease or injury; years lived with disability (YLDs), which quantifies the healthy time lost by a person living with a disability caused by a disease or injury; and disability-adjusted life years (DALYs), a summary measure that is the sum of YLLs and YLDs [17]. These indicators have recently been used to estimate the global burden of diseases and the overall burden of injuries [6]; however—owing especially to the nonavailability of data—studies using them to describe TBI are scarce [12,18].

The aim of this study was to provide an in-depth analysis of the burden of deaths due to TBI by calculating TBI-induced YLLs in 16 European countries in 2013, analyzing their main causes and demographic patterns, using data extracted from death certificates under unified guidelines and collected in a standardized manner.

## Methods

### Study design and setting

A population-wide, cross-sectional epidemiological study was conducted in 16 European countries (Austria, Bulgaria, Croatia, Cyprus, Denmark, Estonia, Hungary, Ireland, Italy, Lithuania, Luxembourg, Romania, Serbia, Slovakia, Slovenia, and United Kingdom) in order to estimate TBI YLLs for the year 2013. The selection of countries was based on the availability of data. The year 2013 was chosen because it was the most recent year for which data were available. The availability of the data in other EU countries for this year was limited because, at the time of this study, not all European countries were submitting data on causes of injury-related deaths in the necessary format (e.g., giving both the external cause and nature of injury) and detail (e.g., giving data in sufficiently small age groups). Thus, the choice was made to use the 16 countries for which data were available, and to extrapolate the findings to the 28 member states of the European Union (EU-28), and this seemed justified under the circumstances.

### Data sources

The data used for all analyses in this study were acquired from the statistical office of the European Union (Eurostat). Eurostat routinely collects data from death certificates from the 28 EU member states, the former Yugoslav Republic of Macedonia, Albania, Iceland, Norway, Liechtenstein, and Switzerland and regularly publishes annual overviews of causes of deaths [19]. For our study, a specifically tailored dataset of micro-level data was provided that, in detail of information, went beyond the regularly published reports. This dataset contained a record for each injury-related death that occurred in the included countries in 2013, where the external cause of death (International Classification of Diseases–10th Revision [ICD-10] codes V01–Y98), the specific nature of injury (ICD-10 codes S00–T98, only 1 diagnosis provided for each record), the age at death, and sex were given. All data used in our analyses were collected at the country level and then—following specific and unified guidelines—submitted to Eurostat, which in turn provided them to us. The study used administratively collected secondary data, and no ethics committee approval was required. No ethics approval was required in order to obtain data from Eurostat.

For the purpose of this study, a TBI-related death was defined as a death where the cause of death was a TBI or a TBI sequela, i.e., from the provided database, records in which the nature of injury was coded as ICD-10 S00–S09 (injuries to the head) or T90 (sequelae of injuries to the head).

### Variable definitions

The European Union life table published by Eurostat was used to determine the life expectancy at death for each recorded death [20]. The number of YLLs for each death was calculated by subtracting the age at death from the life expectancy at the age of death, and summarized using this formula:

$$\mathrm{YLL}=\Sigma \;d_{l}\times e_{l}$$ (1)

where *d*_1_ is the number of fatal cases due to health outcome *l* in a certain period and e_l_ is the expected individual life span at the age of death due to health outcome *l*.

YLLs were summarized into 6 age groups (0–4, 5–14, 15–34, 35–64, 65–84, and ≥85 years) by summing the YLLs in persons in each age group. Crude rates of YLLs were calculated per 100,000 people per year using mid-year populations for each country in total and for the 6 age groups. For comparison purposes (in response to the suggestions of a peer reviewer), number of YLLs and crude TBI YLL rates per 100,000 persons per year are also presented broken down into 5-year age groups (S1 Appendix). In addition, age-standardized rates with 95% CIs were calculated in order to adjust for differences in the age structures of the compared populations (i.e., differences between the analyzed countries). To calculate age-standardized rates, the European standard population was used, which is a theoretical population with its age distribution based on actual age distributions in the populations of the European countries [21]. A pooled estimate was calculated based on age-standardized rates. Further, average YLLs due to TBI per case were calculated with 95% CIs for each country and overall (mean YLLs per case calculated by dividing the sum of YLLs by the number of cases in the respective group or subgroup).

In addition, both numbers of YLLs and YLL rates were stratified by sex, and external cause of injury. Differences between rates (by age group and sex) are presented as rate ratios with 95% CIs.

In order to evaluate the relative importance of TBI in the context of all injuries, proportions of TBI YLLs out of overall injury YLLs were calculated. For this calculation, cases with unspecific codes (e.g., “other and unspecified effects of external causes”); deaths caused by exposure to heat, frost, or intoxication (T15–T65); and cases with other generalized causes (T66–T78; T80–T88) were excluded. Deaths with these causes are either not directly comparable to deaths due to TBI—because the circumstances or mechanisms leading to death are substantially different (e.g., traffic injury versus frostbite)—or the true cause of death is actually unknown and inclusion could bias the calculated proportions by increasing the denominator. Thus, only the following codes were used in the denominator: injuries to the head (S00–S09); injuries involving multiple body regions (T00–T07); injuries to unspecified trunk, limb, or body region (T08–T14); certain early complications of trauma (T79); and sequelae of injuries, of poisoning, and of other consequences of external causes (T90–T98). In order to allow for comparisons, S1–S3 Tables present these analyses with all injury-related deaths (no exclusions) used as the denominator.

The total number of TBI YLLs in the EU was estimated by extrapolating the pooled crude rate of YLLs in the 16 analyzed countries to the EU-28 population count published by Eurostat [22].

### Statistical methods

Pooled analyses were performed in order to estimate summary age-standardized rates of YLLs. In order to model possible heterogeneity of rates in the different countries, the random effects model was applied [23] by the DerSimonian and Laird method, in line with previous studies [5,24]. To assess the heterogeneity of the pooled estimations, *I*^2^ values with 95% confidence intervals were calculated. To make our findings as comparable as possible, in S4 Table we provide pooled rates calculated by applying the fixed effects model (in response to the suggestions of a peer reviewer). The original analysis plan for the study is reported in S1 Text.

## Results

**Fig 1:**
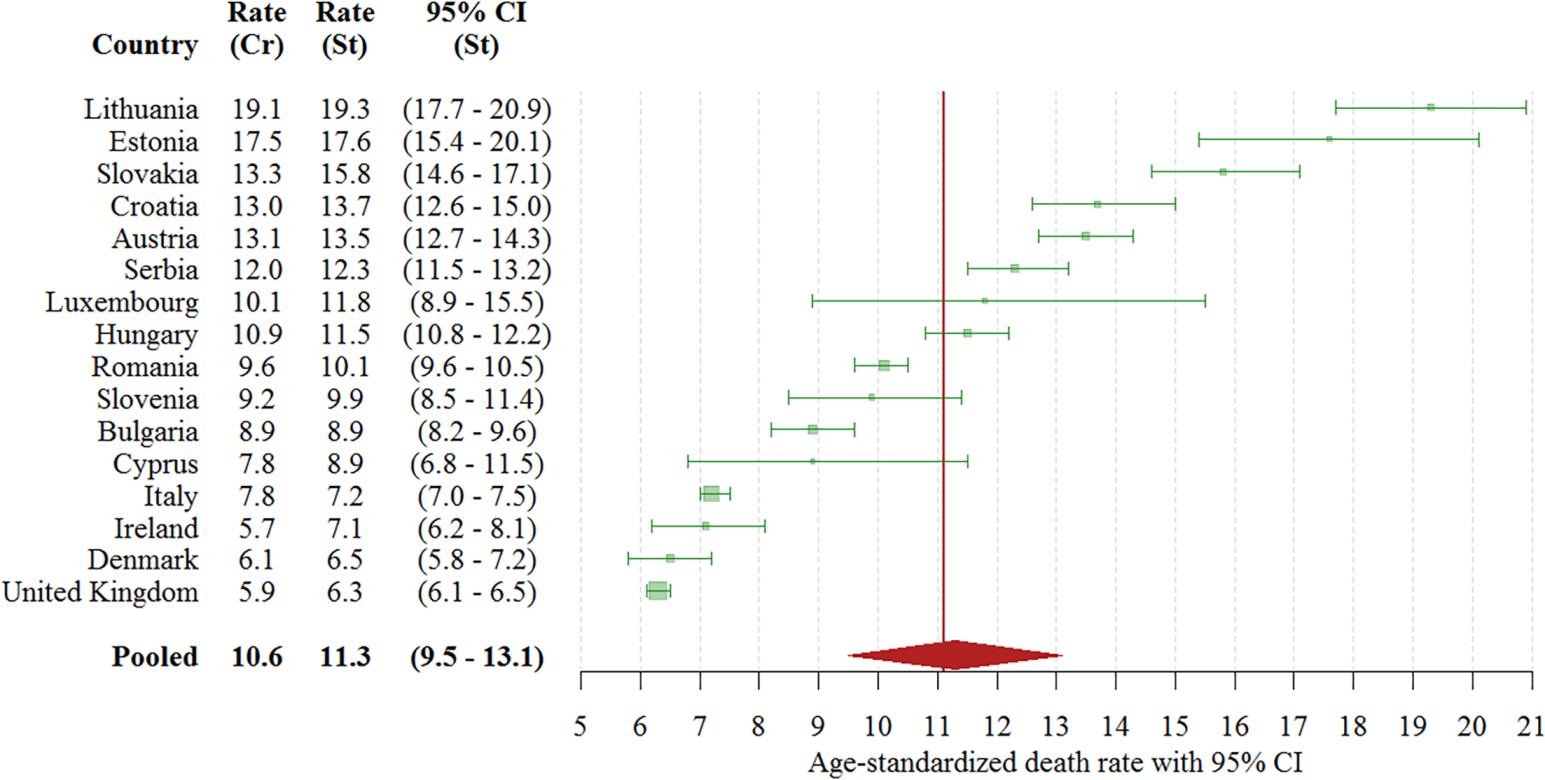
Crude TBI death rates and age-standardized TBI death rates per 100,000 persons in 16 European countries in 2013, with a pooled age-standardized death rate. St, standardized; CI, confidence interval; Cr, crude; TBI, traumatic brain injury. Meta-analysis heterogeneity: *I^2^* = 100% (95% CI: 100% to 100%).

**Fig 2:**
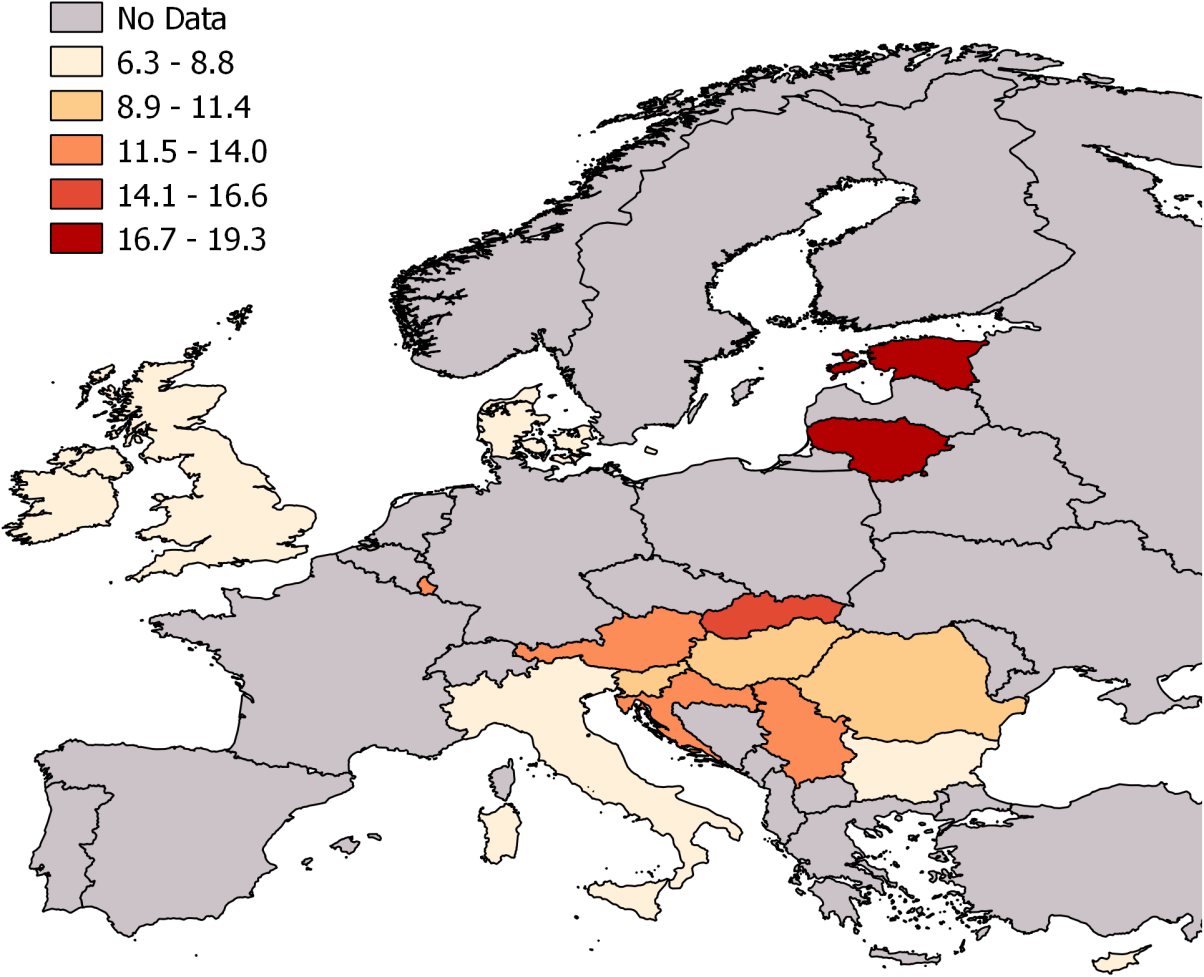
Age-standardized TBI death rates per 100,000 persons in 16 European countries in 2013. TBI, traumatic brain injury. Grey areas were not included in this study.

Based on the data we obtained and the case ascertainment defined in this study, 17,049 TBI deaths were identified in the 16 analyzed countries in 2013, which translates to an age-standardized pooled rate of 11.3 (95% CI: 9.5–13.1) TBI deaths per 100,000 persons per year (Fig 1). Of these deaths, 11,944 (70%) were males. In males, the majority of deaths (8,595, 72%) occurred in persons 35–84 years old, whereas in females most deaths occurred in persons 65 years old and older (3,703, 73%). Fig 2 shows a map with levels of TBI death rates in the analyzed countries. The highest TBI death rates overall and for males were observed in Lithuania, and for females in Austria (consult S5 and S6 Tables for details). The largest sex differences in age-standardized TBI death rates were in Estonia (male to female ratio of 5.8) and Bulgaria (ratio of 4.5), whereas the smallest such differences were in the United Kingdom (ratio of 1.7) and Italy (ratio of 2.1)—consult S7 Table for details.

In order to present the overall magnitude of the problem in the analyzed countries, in Table 1 we present the overall numbers of TBI YLLs by country, age, and sex, along with the size of the population in each country. A total of 374,636 YLLs were attributed to TBI in the 16 analyzed countries in 2013. Of these, 282,870 (76%) were YLLs in the male population. The highest number of TBI YLLs for both sexes was observed in Italy (67,809 in males and 24,481 in females). For males, the lowest number of YLLs was in Luxembourg (1,031), and for females in Cyprus (212). Deaths occurring in the age group 15–64 years old were the largest contributors to TBI YLLs—they caused 73% (274,409) of all TBI YLLs (this pattern was present in both sexes). The sums of the TBI YLLs in the countries are proportional to the population and are higher in more populated countries, where populations at risk are larger. In order to put the numbers of YLLs in context with the population and to compare them more validly, we present crude and age-standardized rates in Table 2.

**Table 1:** Numbers of TBI YLLs in 16 European countries in 2013 by age, sex, and country.

Subgroup	Country	Population	Age group (years)						Total
			0–4	5–14	15–34	35–64	65–84	85+	
**Total**	Italy	60,233,948	1,756	2,007	32,283	32,935	20,029	3,280	92,290
	United Kingdom	64,128,226	2,055	1,270	19,851	26,924	15,036	3,159	68,295
	Romania	19,983,693	2,440	2,343	15,099	27,388	7,307	333	54,910
	Hungary	9,893,082	477	358	6,157	12,314	4,758	479	24,543
	Serbia	7,164,132	310	1,066	7,964	9,760	3,674	127	22,901
	Austria	8,479,375	313	476	4,176	7,785	5,376	876	19,002
	Bulgaria	7,265,115	860	613	5,669	8,428	2,607	125	18,302
	Slovakia	5,413,393	78	714	4,166	9,050	3,050	230	17,288
	Lithuania	2,957,689	83	289	3,831	8,810	2,067	122	15,202
	Croatia	4,254,475	307	280	3,572	5,678	2,373	270	12,480
	Denmark	5,614,932	243	493	2,771	3,444	1,389	151	8,491
	Ireland	4,598,294	160	489	2,905	2,703	927	113	7,297
	Estonia	1,317,997	323	214	2,056	3,558	746	30	6,927
	Slovenia	2,059,953	0	0	836	1,500	910	135	3,381
	Cyprus	861,939	0	0	1,215	570	219	18	2,022
	Luxembourg	543,360	0	65	426	585	202	27	1,305
	Total		9,405 (3%)	10,677 (3%)	112,977 (30%)	161,432 (43%)	70,670 (19%)	9,475 (3%)	374,636
**Males**	Italy	29,187,081	689	1,345	25,902	25,858	12,632	1,383	67,809
	United Kingdom	31,543,582	986	964	14,839	20,219	8,644	1,433	47,085
	Romania	9,753,766	1,293	1,157	12,158	23,679	5,151	217	43,655
	Hungary	4,709,672	311	133	4,892	9,704	3,155	208	18,403
	Serbia	3,488,436	227	402	6,422	7,920	2,580	73	17,624
	Bulgaria	3,535,009	530	539	4,668	7,279	1,828	84	14,928
	Austria	4,139,629	149	337	3,319	6,435	3,738	387	14,365
	Slovakia	2,637,520	78	346	3,446	7,820	2,123	116	13,929
	Lithuania	1,362,443	0	136	3,366	7,218	1,518	38	12,276
	Croatia	2,053,116	226	130	3,000	4,879	1,585	115	9,935
	Denmark	2,785,566	76	195	1,957	2,772	930	64	5,994
	Estonia	615,543	77	65	1,926	3,122	560	14	5,764
	Ireland	2,275,508	77	414	2,426	2,160	482	38	5,597
	Slovenia	1,019,968	0	0	718	1,271	616	60	2,665
	Cyprus	419,287	0	0	1,215	442	146	7	1,810
	Luxembourg	271,765	0	65	314	492	144	16	1,031
	Total		4,719 (2%)	6,228 (2%)	90,568 (32%)	131,270 (46%)	45,832 (16%)	4,253 (2%)	282,870
**Females**	Italy	31,046,867	1,067	662	6,381	7,077	7,397	1,897	24,481
	United Kingdom	32,584,644	1,069	306	5,012	6,705	6,392	1,726	21,210
	Romania	10,229,927	1,147	1,186	2,941	3,709	2,156	116	11,255
	Hungary	5,183,410	166	225	1,265	2,610	1,603	271	6,140
	Serbia	3,675,697	83	664	1,542	1,840	1,094	54	5,277
	Austria	4,339,746	164	139	857	1,350	1,638	489	4,637
	Bulgaria	3,730,106	330	74	1,001	1,149	779	41	3,374
	Slovakia	2,775,873	0	368	720	1,230	927	114	3,359
	Lithuania	1,595,246	83	153	465	1,592	549	84	2,926
	Croatia	2,201,359	81	150	572	799	788	155	2,545
	Denmark	2,829,366	167	298	814	672	459	87	2,497
	Ireland	2,322,787	83	75	479	543	445	75	1,700
	Estonia	702,454	246	149	130	436	186	16	1,163
	Slovenia	1,039,986	0	0	118	229	294	75	716
	Luxembourg	271,595	0	0	112	93	58	11	274
	Cyprus	442,652	0	0	0	128	73	11	212
	Total		4,686 (5%)	4,449 (5%)	22,409 (24%)	30,162 (33%)	24,838 (27%)	5,222 (6%)	91,766

TBI, traumatic brain injury; YLL, year of life lost.

**Table 2:** Crude and age-standardized TBI YLL rates per 100,000 persons in 16 European countries in 2013 by age and sex.

Subgroup	Country	Age group (years)						Crude rate	Age-standardized rate (95% CI)
		0–4	5–14	15–34	35–64	65–84	85+		
**Total**	Estonia	431.2	161.5	604.9	670.4	349.5	113.3	525.6	518.2 (506.1–530.6)
	Lithuania	54.9	102.4	499.6	725.1	427.4	207.4	514.0	505.4 (497.4–513.5)
	Slovakia	26.8	132.5	265.8	394.5	465.3	347.6	319.4	327.3 (322.3–332.3)
	Serbia	93.5	153.0	446.6	317.2	310.2	138.0	319.7	313.4 (309.3–317.5)
	Croatia	145.1	66.6	333.8	320.0	334.3	396.1	293.3	291.0 (285.9–296.2)
	Romania	248.5	109.7	295.1	323.2	244.9	113.3	274.8	270.7 (268.4–273.0)
	Bulgaria	249.7	94.5	321.3	271.8	202.5	105.0	251.9	247.1 (243.5–250.7)
	Hungary	103.9	36.9	247.0	289.4	308.1	278.2	248.1	245.1 (242.0–248.2)
	Luxembourg	0.0	106.2	292.6	254.7	302.2	288.0	240.2	243.6 (230.4–257.5)
	Austria	78.6	58.0	195.5	217.3	402.5	424.6	224.1	224.3 (221.1–227.5)
	Cyprus	0.0	0.0	448.4	170.9	207.3	162.5	234.6	216.1 (206.5–226.2)
	Slovenia	0.0	0.0	165.6	166.8	287.8	337.9	164.1	164.7 (159.1–170.3)
	Ireland	43.5	76.3	235.2	151.6	182.1	181.0	158.7	163.9 (160.1–167.8)
	Italy	64.3	35.4	250.0	126.2	182.1	179.6	153.2	153.7 (152.7–154.7)
	Denmark	78.8	74.2	201.6	152.8	154.7	131.1	151.2	151.9 (148.7–155.2)
	United Kingdom	51.2	17.4	117.7	108.5	155.6	215.6	106.5	108.5 (107.7–109.3)
	Pooled								259.1 (205.8–312.3)
**Males**	Estonia	200.4	95.4	1,100.3	1,230.8	749.2	263.5	936.5	917.0 (893.1–941.7)
	Lithuania	0.0	94.1	859.4	1,275.0	898.2	281.9	901.0	892.7 (876.7–909.0)
	Slovakia	52.1	124.8	429.7	688.9	829.1	629.7	528.1	555.2 (545.4–565.2)
	Serbia	133.0	112.1	704.0	525.1	508.7	230.1	505.2	492.8 (485.5–500.3)
	Croatia	207.5	60.3	549.4	556.7	550.8	631.3	483.9	483.7 (474.0–493.7)
	Romania	256.2	105.5	461.1	564.1	422.4	220.6	447.6	440.8 (436.7–445.1)
	Bulgaria	299.5	161.5	511.7	472.0	345.0	208.2	422.3	410.6 (404.0–417.3)
	Hungary	131.8	26.8	384.2	469.8	532.8	458.6	390.8	394.1 (388.2–400.0)
	Luxembourg	0.0	207.2	424.8	419.4	469.9	566.1	379.2	388.6 (364.5–414.6)
	Cyprus	0.0	0.0	900.8	278.6	297.2	169.2	431.9	383.8 (365.7–402.9)
	Austria	72.8	80.0	306.6	361.7	629.0	665.3	347.0	356.1 (350.2–362.1)
	Slovenia	0.0	0.0	273.9	276.4	459.1	620.0	261.3	271.2 (260.7–282.3)
	Ireland	41.1	126.3	397.4	243.6	198.4	189.1	245.9	248.4 (241.8–255.3)
	Italy	49.1	46.1	395.3	201.4	257.0	246.5	232.3	233.8 (232.1–235.6)
	Denmark	48.0	57.4	280.4	245.3	220.4	173.7	215.2	217.1 (211.7–222.8)
	United Kingdom	48.0	25.8	174.6	164.9	192.1	290.5	149.3	153.8 (152.4–155.3)
	Pooled								427.5 (290.0–564.9)
**Females**	Lithuania	113.2	111.5	123.8	245.3	174.4	185.8	183.4	181.3 (174.8–188.0)
	Estonia	673.1	232.1	78.5	157.2	133.8	75.1	165.3	166.3 (156.9–176.3)
	Serbia	51.4	196.3	177.0	117.3	161.6	90.3	143.6	143.9 (140.0–147.9)
	Slovakia	0.0	140.4	94.0	106.2	232.1	238.6	121.0	126.4 (122.1–130.8)
	Hungary	74.2	47.7	103.7	119.2	168.4	214.0	118.5	116.1 (113.2–119.0)
	Croatia	78.5	73.4	109.1	88.9	186.8	309.5	115.6	113.7 (109.3–118.2)
	Romania	240.3	114.2	118.6	86.8	122.2	59.2	110.0	110.4 (108.4–112.5)
	Austria	84.8	34.8	81.3	74.9	221.0	329.9	106.8	103.7 (100.7–106.8)
	Luxembourg	0.0	0.0	155.9	82.7	159.1	162.7	100.6	102.0 (90.2–115.1)
	Bulgaria	197.0	23.4	117.5	73.7	102.8	51.6	90.4	89.2 (86.2–92.3)
	Denmark	111.0	91.7	120.3	59.8	96.5	110.6	88.2	87.9 (84.5–91.4)
	Ireland	45.8	23.8	76.7	60.6	167.0	176.6	73.1	80.7 (76.8–84.7)
	Italy	80.4	24.1	100.3	53.4	121.5	150.0	78.9	76.8 (75.8–77.8)
	Slovenia	0.0	0.0	48.5	52.0	161.3	248.1	68.7	66.3 (61.5–71.4)
	United Kingdom	54.6	8.6	59.9	53.4	123.8	177.6	65.1	65.2 (64.3–66.0)
	Cyprus	0.0	0.0	0.0	72.9	129.5	170.1	47.9	55.8 (48.4–64.1)
	Pooled								105.4 (89.1–121.6)

Meta-analysis heterogeneity: *I^2^* for total = 100% (95% CI: 100% to 100%); *I^2^* for males = 100% (95% CI: 100% to 100%); *I^2^* for females = 100% (95% CI: 100% to 100%). TBI, traumatic brain injury; YLL, year of life lost.

**Fig 3:**
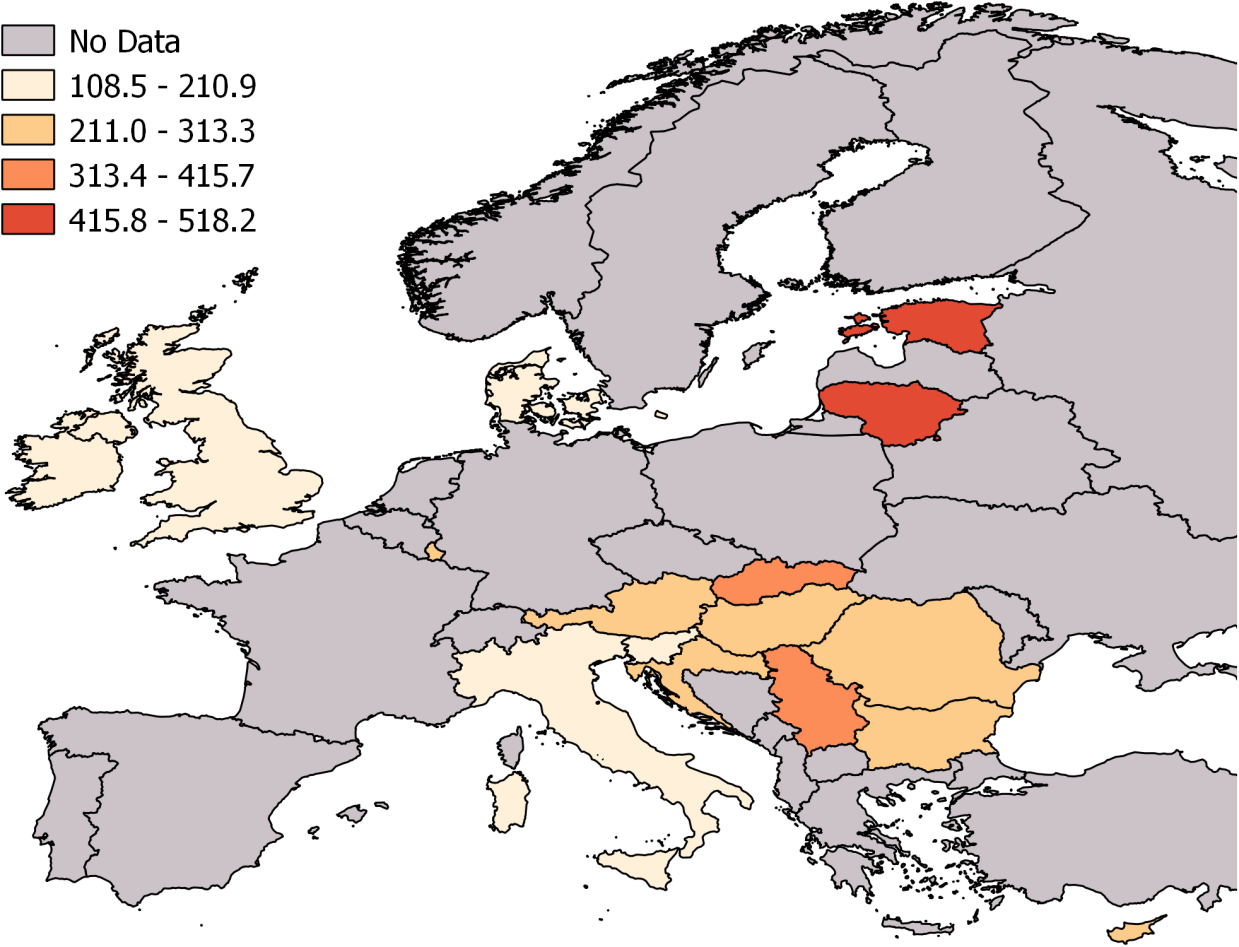
Age-standardized TBI YLL rates per 100,000 persons in 16 European countries in 2013. TBI, traumatic brain injury; YLL, year of life lost. Grey areas were not included in this study.

Crude rates of TBI YLLs are presented for each age group, along with the overall crude rate and the age-standardized rate per 100,000 persons per year—by country and sex. The highest age-standardized rates overall and in males were found for Estonia (518.2 [95% CI: 506.1 to 530.6] and 917.0 [95% CI: 893.1 to 941.7], respectively), and in females for Lithuania (181.3 [95% CI: 174.8 to 188.0]). The geographical distribution of TBI YLLs is presented in Fig 3. In S8 Table, age differences in TBI YLLs are presented as rate ratios with 95% CIs. In both sexes, and overall, compared to the reference category (35–64 years), the highest rates are in the age group 15–34 years (rate ratios of 1.18 overall, 1.13 in males and 1.29 in females). While in males the rates in the age groups 65–84 and ≥85 years are similar to that of the reference category (rate ratios of 1.0 and 0.93), in females the rates in the older groups are substantially higher (rate ratio of 1.92 for the age group 65–84 years and 2.34 for the age group ≥85 years), confirming the shift of TBI to higher ages in the female population.

In the same manner, sex differences are presented as rate ratios by country in S9 Table. Overall, the male to female rate ratio was 3.24 (95% CI: 3.22 to 3.27), ranging from 2.29 (95% CI: 2.26 to 2.33) in the United Kingdom to 9.01 (95% CI: 7.83 to 10.41) in Cyprus. The pooled age-standardized TBI YLL rates for the 16 countries were 259.1 (95% CI: 205.8 to 312.3) per 100,000 persons per year overall, 427.5 (95% CI: 290.0 to 564.9) in males, and 105.4 (95% CI: 89.1 to 121.6) in females.

Mean YLLs per TBI death was calculated in order to describe the burden presented by each death. Table 3 presents the findings for this measure in detail by country, age, and sex. On average, 1 TBI-related death translated into 24.3 (95% CI: 22.0 to 26.6) YLLs overall, 25.6 (95% CI: 23.4 to 27.8) YLLs in males, and 20.9 (95% CI: 17.9 to 24.0) YLLs in females. In general, per-case YLLs decreased with increasing age: from 79.3 YLLs/case in the age group 0–4 years to 3.4 in the age group 85 years and older, with corresponding values of 76.3 and 3.3 in males and 82.3 and 3.8 in females, respectively.

**Table 3:** Burden of TBI YLLs per death in 16 European countries in 2013 by age and sex.

Subgroup	Country	Age group (years)						Total (95% CI)
		0–4	5–14	15–34	35–64	65–84	85+	
**Total**	Cyprus	—	—	55.2	28.5	12.2	2.6	30.2
	Estonia	80.8	71.3	52.7	29.4	13.6	3.8	30.1
	Romania	78.7	71.0	54.7	29.7	12.7	3.8	28.5
	Bulgaria	78.2	68.1	54.0	29.5	12.5	4.2	28.2
	Ireland	80.0	69.9	53.8	30.0	12.5	3.2	27.9
	Lithuania	83.0	72.3	53.2	30.0	12.5	4.2	26.9
	Serbia	77.5	71.1	53.4	28.5	11.7	3.5	26.7
	Denmark	81.0	70.4	56.6	30.5	12.2	2.6	24.7
	Slovakia	78.0	71.4	53.4	28.2	12.3	3.8	24.1
	Luxembourg	—	65.0	53.3	30.8	11.9	2.7	23.7
	Hungary	79.5	71.6	54.0	28.5	12.2	3.7	22.8
	Croatia	76.8	70.0	55.0	28.1	11.4	4.0	22.6
	Italy	79.8	69.2	54.3	31.0	11.0	2.8	19.7
	United Kingdom	79.0	70.6	55.0	30.6	11.2	2.8	18.2
	Slovenia	—	—	52.3	27.3	11.1	3.8	17.9
	Austria	78.3	68.0	54.2	28.4	11.4	3.1	17.0
	Average	79.3	70.0	54.1	29.3	12.0	3.4	24.3 (22.0 to 26.6)
**Males**	Cyprus	—	—	55.2	27.6	12.2	1.5	32.9
	Ireland	77.0	68.9	52.7	29.2	12.4	2.7	31.1
	Estonia	77.0	65.1	52.0	28.9	13.3	4.8	30.0
	Bulgaria	75.7	67.4	53.1	29.1	12.2	3.8	28.4
	Romania	76.1	68.0	53.6	29.3	12.2	3.9	28.2
	Lithuania	—	68.1	52.6	29.5	12.3	3.4	27.6
	Serbia	75.7	66.9	52.2	27.7	11.3	3.2	26.3
	Denmark	76.0	65.1	54.4	29.8	12.1	2.5	25.4
	Luxembourg	—	65.1	52.3	30.8	12.0	2.6	25.1
	Slovakia	77.7	69.1	52.2	27.5	12.4	3.4	24.8
	Croatia	75.3	65.1	53.6	27.7	11.2	3.7	24.2
	Hungary	77.7	66.6	53.2	27.8	12.1	3.7	24.1
	Italy	76.5	67.3	53.3	30.2	10.7	2.6	22.0
	Slovenia	—	—	51.3	26.5	11.0	4.6	20.3
	United Kingdom	75.9	68.9	53.4	29.6	10.6	2.7	20.1
	Austria	74.6	67.3	53.5	27.5	11.1	3.1	18.8
	Average	76.3	67.1	53.0	28.7	11.8	3.3	25.6 (23.4 to 27.8)
**Females**	Estonia	81.8	74.7	64.8	33.5	14.3	3.2	30.6
	Romania	81.9	74.1	60.0	32.3	13.8	3.7	29.5
	Serbia	82.6	73.8	59.3	32.9	12.9	4.2	27.8
	Bulgaria	82.5	73.7	58.9	31.9	13.2	5.1	27.0
	Lithuania	83.3	76.7	58.1	32.5	13.1	4.7	24.4
	Denmark	83.3	74.4	62.6	33.6	12.4	2.7	23.1
	Slovakia	—	73.7	60.0	33.3	12.2	4.2	21.4
	Ireland	82.6	74.7	59.9	33.9	12.7	3.6	20.7
	Hungary	83.0	75.0	57.5	31.4	12.3	3.7	19.6
	Luxembourg	—	—	55.9	31.0	11.5	2.7	19.5
	Croatia	80.6	75.2	63.5	30.7	11.8	4.3	18.0
	Cyprus	—	—	—	31.9	12.2	5.7	17.7
	Italy	82.1	73.6	59.1	34.2	11.6	3.1	15.3
	United Kingdom	82.3	76.6	60.4	34.0	12.2	2.9	15.0
	Austria	82.0	69.7	57.1	33.7	12.2	3.1	13.2
	Slovenia	—	—	58.8	32.6	11.3	3.3	12.3
	Average	82.3	74.3	59.7	32.7	12.5	3.8	20.9 (17.9 to 24.0)

TBI, traumatic brain injury; YLL, year of life lost.

**Fig 4:**
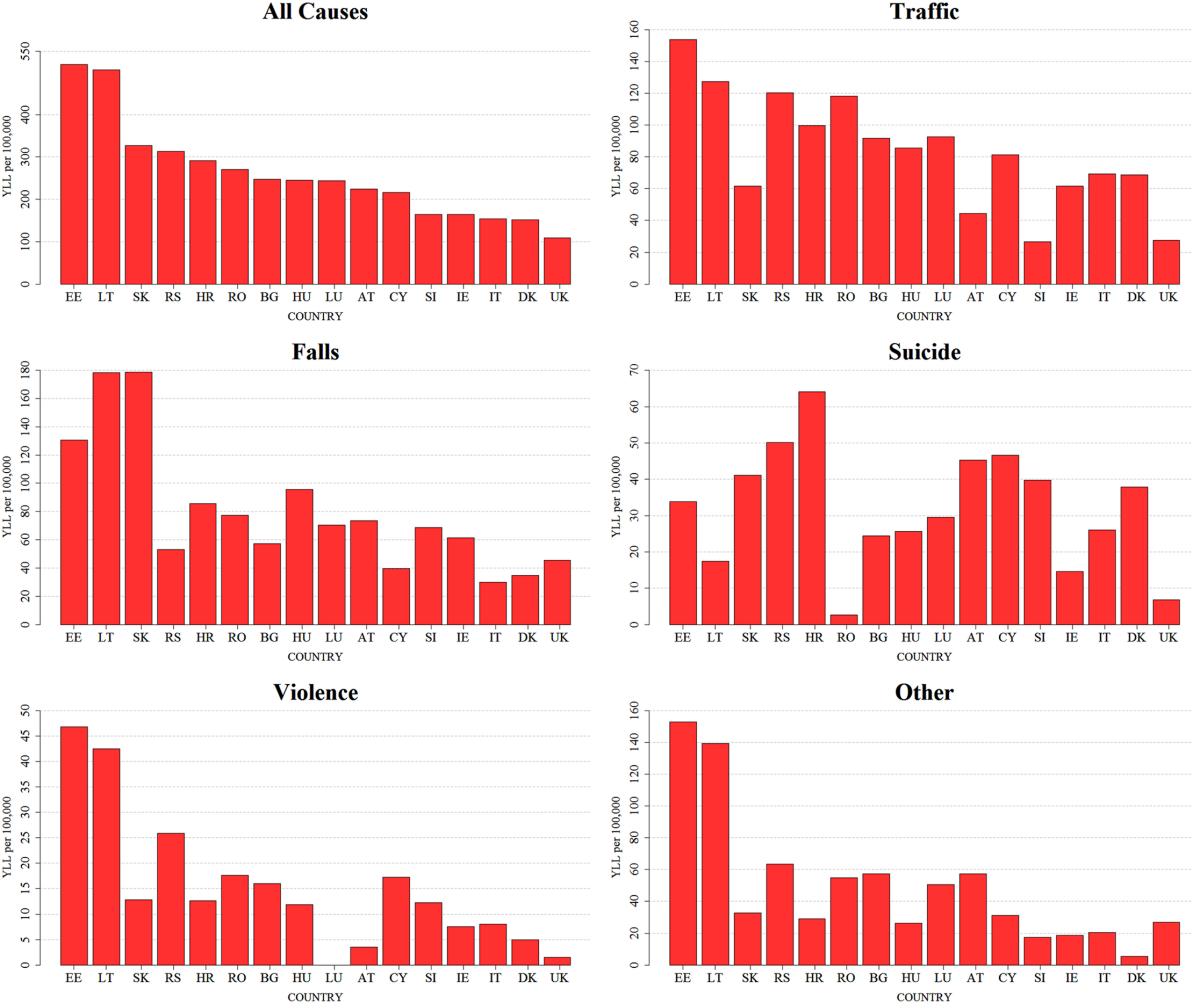
Age-standardized TBI YLL rates per 100,000 persons by external cause of death in 16 European countries in 2013. The scaling of the sub-figures has been specifically adapted for each group of causes to better show the variation between the countries. AT, Austria; BG, Bulgaria; CY, Cyprus; DK, Denmark; EE, Estonia; HR, Croatia; HU, Hungary; IE, Ireland; IT, Italy; LT, Lithuania; LU, Luxembourg; RO, Romania; RS, Serbia; SI, Slovenia; SK, Slovakia; TBI, traumatic brain injury; UK, United Kingdom; YLL, year of life lost.

Falls and traffic injuries were the most common causes of TBI YLLs across the 16 countries—as presented in Fig 4—followed by suicide, violence, and other causes. After excluding deaths caused by natural forces, intoxication, and other generalized or unknown causes (see Methods), a total of 991,420 injury YLLs were identified overall, of which 714,757 (72%) were in males (S10 Table). These translated into pooled age-standardized rates of 627.9 (95% CI: 522.9 to 733.0) YLLs per 100,000 persons per year overall, 956.6 (95% CI: 782.6 to 1,130.6) in males and 318.9 (95% CI: 271.1 to 366.6) in females (S11 Table).

**Fig 5:**
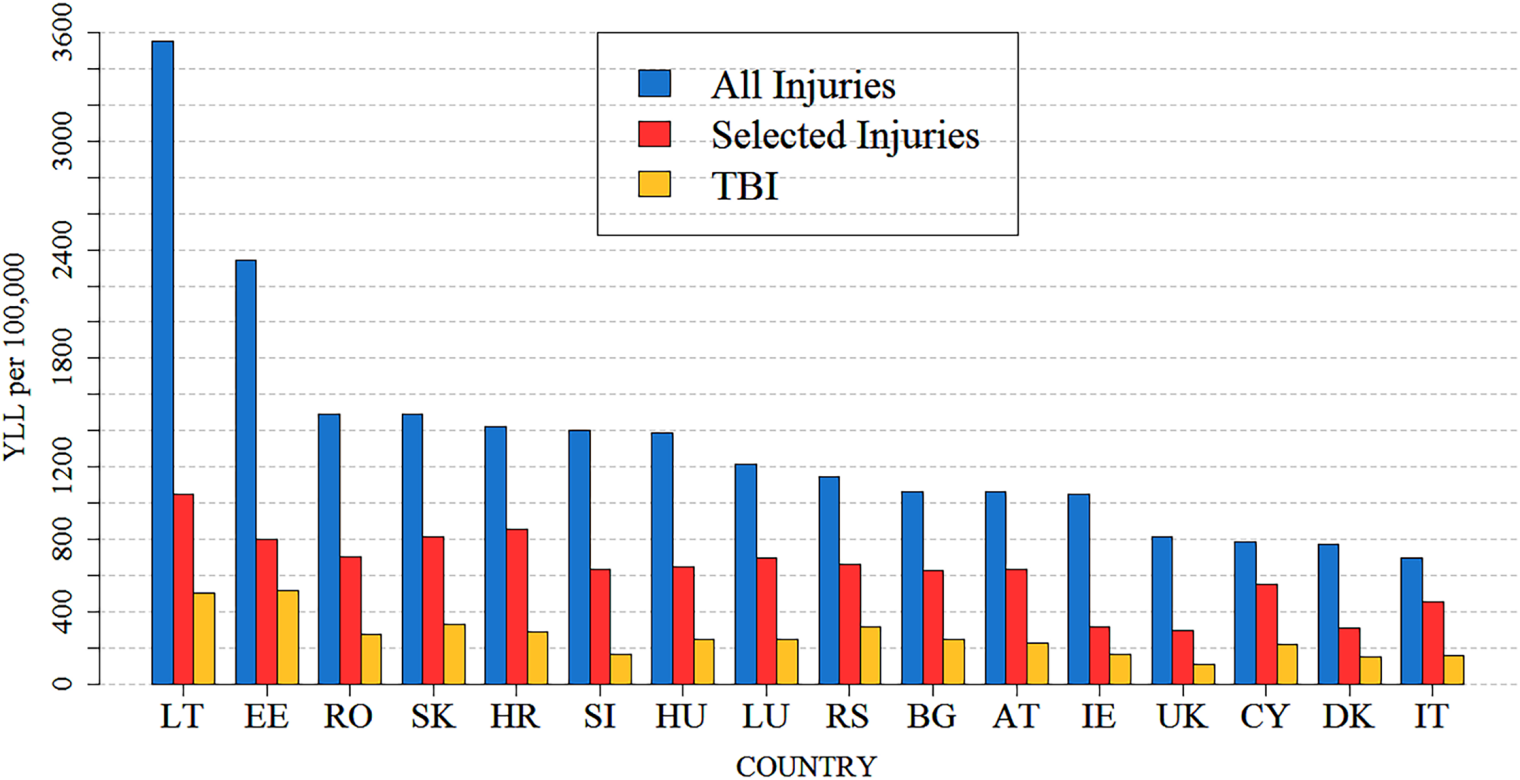
Age-standardized injury YLL rates and TBI YLL rates per 100,000 persons in 16 European countries in 2013. “Selected Injuries” includes the following causes of death: injuries to the head (S00–S09); injuries involving multiple body regions (T00–T07); injuries to unspecified trunk, limb, or body region (T08–T14); certain early complications of trauma (T79); and sequelae of injuries, of poisoning, and of other consequences of external causes (T90–T98). For sex-specific data see S1 Fig. AT, Austria; BG, Bulgaria; CY, Cyprus; DK, Denmark; EE, Estonia; HR, Croatia; HU, Hungary; IE, Ireland; IT, Italy; LT, Lithuania; LU, Luxembourg; RO, Romania; RS, Serbia; SI, Slovenia; SK, Slovakia; TBI, traumatic brain injury; UK, United Kingdom; YLL, year of life lost.

TBI YLLs as a proportion of overall injury YLLs are presented in Fig 5 in order to indicate their relative importance (see S1 Fig for sex-specific data). After excluding deaths caused by natural forces, intoxication, and other generalized or unknown causes, TBIs contributed on average 41% (44% in males and 34% in females) of all injury YLLs—with the highest contributions of TBI YLLs in both sexes in the age group 0–4 years (56% in males and 69% in females) (see S12 Table). For comparison purposes, TBI-related YLLs as a proportion of all injury YLLs are also presented without excluding deaths caused by natural forces, intoxication, and other generalized or unknown causes (see S13–S15 Tables).

In order to provide an estimation of TBI-related YLLs for the EU, the pooled crude rates from our study were extrapolated to the population of the 28 EU member states. These findings are presented in Table 4: based on our pooled rates, 1,319,496 (95% CI: 1,043,675 to 1,595,317) YLLs were attributable to TBI in the EU-28 in 2013 overall, with 1,058,962 (95% CI: 698,748 to 1,419,177) in males and 271,203 (95% CI: 227,211 to 315,196) in the female population.

**Table 4:** Estimated numbers of YLLs in the EU-28 based on extrapolation of pooled crude YLL rates.

Subgroup	Population	Pooled crude rate	Estimated YLLs (95% CI)
Total	505,166,839	261.2 (206.6 to 315.8)	1,319,496 (1,043,675 to 1,595,317)
Males	246,384,899	429.8 (283.6 to 576.0)	1,058,962 (698,748 to 1,419,177)
Females	258,781,940	104.8 (87.8 to 121.8)	271,203 (227,211 to 315,196)

EU-28, 28 member states of the European Union; TBI, traumatic brain injury; YLL, year of life lost.

## Discussion

### Main findings

We conducted a large-scale, cross-sectional, population-based analysis of YLLs due to TBI in 16 European countries for the year 2013. We found that in the selected countries a total of 17,049 TBI-related deaths occurred in 2013. These translated into a total of 374,636 YLLs. The pooled age-standardized rates of YLLs per 100,000 were 259.1 (95% CI: 205.8 to 312.3) overall, 427.5 (95% CI: 290.0 to 564.9) in males, and 105.4 (95% CI: 89.1 to 121.6) in females. Males contributed more substantially to the overall numbers of YLLs than females (282,870 YLLs, 76% of all TBI YLLs), which translated into a rate ratio of 3.24 (95% CI: 3.22 to 3.27). Each TBI death was on average associated with 24.3 (95% CI: 22.0 to 26.6) YLLs overall, 25.6 (95% CI: 23.4 to 27.8) in males and 20.9 (17.9 to 24.0) in females. Falls and traffic injuries were the most common external causes of TBI. TBI contributed on average 41% (44% in males and 34% in females) to overall injury YLLs in the 16 countries. Extrapolating our findings, about 1.3 million YLLs were attributable to TBI in the EU-28 in 2013 overall, 1.1 million in males, and 270,000 in females. To our knowledge, this is the largest and most comprehensive analysis of TBI YLLs in Europe to date.

### Interpretation and generalizability

For all our analyses, microdata on causes of death obtained from Eurostat were used. Eurostat collects data on causes of death from countries, which extract them from death certificates in accordance with EU Commission Regulation No 328/2011 on community statistics on public health and health and safety at work [25]. This regulation defines the scope, provides definitions of variables and characteristics of the data, and aims to achieve the highest possible degree of harmonization and comparability of the information obtained from various countries [26]. Thus, to the best of our knowledge, for our study we have used the most valid and comparable data that were available—as such, we believe that the information and comparisons presented in this paper are valid. However, the relatively large between-country differences in YLLs suggest that there still may be factors beyond true country variability affecting the size of this variation. In general, countries follow ICD-10 standards, making the data collection procedures on causes of death relatively homogenous; however, factors such as differences in interpretation and use of ICD-10 rules at the national level, nonapplication of WHO updates, and differences in reporting of deaths of residents abroad and deaths of nonresidents in the reporting country may hinder the general comparability of the data on causes of death [26], and thus the generalizability of our findings.

Besides these systematic factors, country characteristics, such as age distribution and general economy level, may influence the numbers of reported TBI deaths. A recent study that evaluated TBI-related mortality in 25 European countries found that countries with higher gross domestic product tended to have higher TBI death rates. Furthermore, this study reports that the substantial between-country differences in TBI death rates could be driven by varying degrees of attributing death to multiple injuries—countries that reported relatively low numbers of TBI-related deaths at the same time reported relatively high numbers of death due to multiple injuries. Thus, a substantial number of TBI deaths may be in some countries “hidden” under multiple injury deaths [5]. These factors may also have influenced the findings of this study, as both studies used data from the same source. In a similar manner, variations in use of “garbage codes” for cause of death (e.g., general causes such as “unknown” or “other”) may have an influence on the between-country variation observed in this study.

Despite all these issues, by using data routinely collected by an official European authority, based on a specific EU regulation, for the same year, and using the same coding system for case ascertainment, our study overcame many limitations of other types of investigations (such as the heterogeneity of time, case ascertainment, and geographical coverage of studies included in systematic reviews of TBI epidemiology [14–16])—which supports the validity and generalizability of our findings.

### Comparison to other studies

To our knowledge, only 2 previous studies have specifically analyzed and reported TBI-related YLLs. A study from the Netherlands [18] reported 118,207 TBI-related YLLs annually for the period of 2010–2012. As data for the Netherlands was not made available by Eurostat for our study, we are not able to directly compare these findings to ours. However, by dividing the number of YLLs reported in the Dutch study by the mean total population of the Netherlands for 2010–2012 according to Eurostat (16,653,712) [22], we were able to obtain a crude YLL rate of 710 per 100,000 persons per year. This rate is higher than that of the highest ranking country in our comparison (Estonia, with a crude TBI-related YLL rate of 525.6). However, it is important to note that the number of deaths in the Dutch study was estimated using average case fatality rates, which further limits the comparability with our findings.

Another study analyzed TBI-related YLLs in New Zealand [12] and reported a total of 14,386 TBI-related YLLs in 2010. Using this value and New Zealand’s 2010 population estimate of 4,353,000 [27] yields a crude rate of TBI-related YLLs of 330.5 per 100,000 persons per year. Such a rate is within the range of crude rates reported for the analyzed countries in our study and is similar to rates we found for Slovakia (319.4), Serbia (319.7), and Croatia (293.3). However, we note that this study used different methods for YLL estimation (e.g., different life table) and a different definition for TBI death, which hinders the general comparability of its findings to ours.

An earlier study assessed the overall burden of injuries in 6 European countries [28]. In both this study and ours, rates of overall injury-related YLLs are reported for Austria, Denmark, and Ireland, and thus can be compared. For Austria the previous study reported 1,710 YLLs per 100,000 persons per year; in our study, the crude rate was 1,078. For Denmark the rates were 1,550 versus 766, and for Ireland the rates were 1,530 versus 1,035. Thus, the rates found in our study are consistently lower, which may be caused by the relatively large time lag between the 2 studies (e.g., 1999 versus 2013).

The pooled age-standardized all injury YLL rate in our study is lower than the global age-standardized YLL rate reported by the Global Burden of Disease Study 2013 [6]—1,355.5 (95% CI: 1,083.8 to 1,627.1) in our study and 2,945 (95% uncertainty interval: 2,796 to 3,129) in the Global Burden of Disease Study 2013 injury study. This might be explained by the fact that the latter study reports a global estimate, and the observed difference may well reflect the patterns of injury deaths globally. On the other hand, the reported global rate falls within the range of age-standardized all injury YLL rates reported in the 16 analyzed countries in our study and is comparable to the rates found in Lithuania (3,554.2 [95% CI: 3,533.0 to 3,575.5]) and Estonia (2,339.1 [95% CI: 2,313.2 to 2,365.1]).

### Implications for policy-making and research

In this study we performed to our knowledge the largest and most comprehensive analysis of TBI-related YLLs in Europe to date. Previous studies relied on analyzing and presenting death rates or case fatality rates in order to describe the magnitude of the burden of fatal TBI for the populations of countries [14–16]. Although important, such analyses do not put the problem of TBI deaths into the broader context of social and economic affairs of the respective countries. Our findings emphasize that with each death, large numbers of years of life are lost in the age groups of economically active people, which underlines the significant burden TBI imposes on the economy of countries and the serious impact on the life of families. We quantified the average number of YLLs due to TBI deaths per 100,000 persons and the average number of YLLs per TBI death in 16 countries, and provided these findings stratified by sex, age, and external cause of injury. We believe that this information could facilitate policy-makers in tailoring preventive action so that actions are targeted to the high-risk populations. Communicating the implications of TBI deaths using YLLs as a measure (rather than numbers of deaths) may help the general public to better grasp the magnitude of the problem, and could help to raise awareness about TBI as a major public health problem in general.

Although YLLs provide a more comprehensive measure of the burden of TBI deaths on a population, they do not capture the burden imposed by nonfatal TBI. Our findings can serve as a basis for analysis of the overall burden of TBI using metrics such as DALYs.

### Limitations

There are some limitations to this study that we would like to acknowledge. The differences in the calculated YLLs and all related variables inherit all possible bias and errors that were present in the raw data provided for us by Eurostat. We were not able to control these, or mitigate them in any way. All interpretations of our findings should be made with this in mind. The extrapolations to the population of the EU-28 are based on 16 countries, and it is possible that they are biased—they should be considered an estimation only. The reason for analyzing data from 16 countries and extrapolating the results to the whole EU-28 instead of using data from all countries was that the data for the rest of the EU countries were not available in the necessary format or with sufficient detail (e.g., countries did not provide the ICD-10 codes for nature of injury along with the ICD-10 codes for external causes, or they provided data grouped into larger age groups). Although this limits the validity of our extrapolations, the approach seemed justified under the circumstances. In order to analyze the full population burden of TBI, nonfatal cases must also be taken into consideration using metrics such as YLDs or DALYs. In this paper we were not able to estimate these, due to the nonavailability of data. Future research should be oriented towards these analyses.

### Conclusions

Our study showed that TBI-related deaths have a substantial impact at the individual and population level in Europe and present an important societal and economic burden that must not be overlooked. We provide information valuable for policy-makers, enabling them to evaluate and plan preventive activities and resource allocation, and to formulate and implement strategic decisions. In addition, our results can serve as a basis for analyzing the overall burden of TBI in the population.

## Supporting information

S1 Appendix Numbers of TBI YLLs and crude TBI YLL rates by age group and sex. (PDF)

S1 Fig Age-standardized injury YLL rates in 16 European countries by sex. (PDF)

S1 GATHER Checklist GATHER checklist. (PDF)

S1 Table Total numbers of injury YLLs in 16 European countries in 2013 by age group and sex (all causes of death included). (PDF)

S2 Table Crude and age-adjusted injury YLL rates in 16 European countries in 2013 by age group and sex (all causes of death included). (PDF)

S3 Table Contribution of TBI YLLs to injury YLLs in 16 European countries in 2013 by age group and sex (all causes of death included). (PDF)

S4 Table Comparison of pooled age-standardized rates of TBI YLLs per 100,000 persons in 16 European countries in 2013 calculated using the random effects model and the fixed effects model. (PDF)

S5 Table Numbers of TBI deaths in 16 European countries in 2013 by age group, country, and sex. (PDF)

S6 Table Crude and age-standardized TBI death rates in 16 European countries in 2013 by age and sex. (PDF)

S7 Table Male to female rate ratios of TBI death rates in 16 European countries in 2013. (PDF)

S8 Table Rate ratios of TBI YLL rates per 100,000 persons in 16 European countries in 2013 by age and sex. (PDF)

S9 Table Male to female rate ratios of TBI YLL rates in 2013 by country. (PDF)

S10 Table Total numbers of injury YLLs in 16 European countries in 2013 by age group and sex. (PDF)

S11 Table Crude and age-adjusted injury YLL rates in 16 European countries by age group and sex. (PDF)

S12 Table Contribution of TBI YLLs to injury YLLs by country, age group, and sex. (PDF)

S13 Table Total numbers of injury YLLs in 16 European countries in 2013 by age group and sex (all causes of death included). (PDF)

S14 Table Crude and age-adjusted injury YLL rates in 16 European countries in 2013 by age group and sex (all causes of death included). (PDF)

S15 Table Contribution of TBI YLLs to injury YLLs in 16 European countries in 2013 by age group and sex (all causes of death included). (PDF)

S1 Text Original analysis plan of the study. (PDF)

## Abbreviations

DALY: disability-adjusted life year
EU-28: 28 member states of the European Union
ICD-10: International Classification of Diseases–10th Revision
TBI: traumatic brain injury
YLD: year lived with disability
YLL: year of life lost
