# Supplementary material for: Years of life lost due to traumatic brain injury in Europe: A cross-sectional analysis of 16 countries
Source: PLoS Med. 2017 Jul 11;14(7):e1002331. doi: 10.1371/journal.pmed.1002331 (PMC5507416; doi:10.1371/journal.pmed.1002331)
Supplement: S1 Appendix — (PDF) [file pmed.1002331.s001.pdf]

**S1 Appendix. Numbers of TBI YLLs and crude TBI YLL rates by age group and sex.**

**Table-A: Numbers of TBI YLLs in 16 European countries in 2013 by age group, for both sexes combined**

| Country/ Age group | 0-4 | 5-9 | 10-14 | 15-19 | 20-24 | 25-29 | 30-34 | 35-39 | 40-44 | 45-49 | 50-54 | 55-59 | 60-64 | 65-69 | 70-74 | 75-79 | 80-84 | 85 + | Total |
|---|---|---|---|---|---|---|---|---|---|---|---|---|---|---|---|---|---|---|---|
| **Austria** | 313 | 332 | 865 | 1202 | 992 | 1115 | 676 | 953 | 1690 | 1726 | 1435 | 143 | 1307 | 1114 | 1851 | 1203 | 1208 | 876 | **19001** |
| **Bulgaria** | 860 | 402 | 926 | 2130 | 1203 | 1410 | 1506 | 1549 | 1257 | 1291 | 1286 | 211 | 1537 | 1045 | 595 | 561 | 406 | 125 | **18300** |
| **Croatia** | 307 | 130 | 1240 | 807 | 828 | 698 | 728 | 824 | 816 | 876 | 1338 | 150 | 1095 | 472 | 648 | 745 | 508 | 270 | **12480** |
| **Cyprus** | 0 | 0 | 419 | 450 | 209 | 137 | 86 | 115 | 103 | 53 | 25 | 0 | 188 | 82 | 54 | 43 | 42 | 18 | **2024** |
| **Denmark** | 243 | 265 | 1007 | 875 | 367 | 521 | 468 | 725 | 661 | 754 | 419 | 228 | 417 | 481 | 343 | 294 | 273 | 151 | **8492** |
| **Estonia** | 323 | 139 | 368 | 624 | 410 | 654 | 433 | 599 | 664 | 796 | 565 | 76 | 502 | 305 | 223 | 194 | 25 | 30 | **6930** |
| **Hungary** | 477 | 207 | 1176 | 1719 | 1877 | 1385 | 1740 | 1620 | 1888 | 1975 | 2896 | 151 | 2196 | 1422 | 1462 | 1038 | 837 | 479 | **24545** |
| **Ireland** | 160 | 128 | 368 | 1097 | 743 | 697 | 347 | 533 | 432 | 533 | 549 | 360 | 308 | 347 | 214 | 183 | 183 | 113 | **7295** |
| **Italy** | 1756 | 1279 | 6991 | 9739 | 8313 | 7239 | 6329 | 6609 | 6258 | 5390 | 4110 | 729 | 4239 | 4250 | 4526 | 5920 | 5333 | 3280 | **92290** |
| **Lithuania** | 83 | 66 | 608 | 1139 | 1000 | 1084 | 1177 | 1604 | 1540 | 1943 | 1497 | 223 | 1049 | 738 | 590 | 516 | 225 | 122 | **15204** |
| **Luxembourg** | 0 | 65 | 61 | 55 | 214 | 95 | 42 | 151 | 163 | 57 | 150 | 0 | 22 | 49 | 54 | 67 | 31 | 27 | **1303** |
| **Romania** | 2440 | 1225 | 3919 | 4338 | 3760 | 3082 | 4481 | 5127 | 4997 | 3759 | 4763 | 1118 | 4262 | 2625 | 2301 | 1505 | 877 | 333 | **54912** |
| **Serbia** | 310 | 544 | 1286 | 2411 | 2043 | 2224 | 1196 | 1577 | 1779 | 1516 | 1600 | 521 | 2092 | 993 | 873 | 1193 | 615 | 127 | **22900** |
| **Slovakia** | 78 | 342 | 691 | 1210 | 990 | 1275 | 1016 | 1218 | 1713 | 1564 | 1836 | 371 | 1703 | 974 | 908 | 687 | 481 | 230 | **17287** |
| **Slovenia** | 0 | 0 | 0 | 396 | 102 | 338 | 126 | 266 | 108 | 256 | 414 | 0 | 329 | 151 | 279 | 246 | 235 | 135 | **3381** |
| **United Kingdom** | 2055 | 398 | 4547 | 6354 | 4998 | 3953 | 3887 | 4893 | 5267 | 5895 | 3983 | 873 | 3000 | 3890 | 3123 | 3773 | 4249 | 3159 | **68297** |
| **Total** | **9405** | **5522** | **24472** | **34546** | **28049** | **25907** | **24238** | **28363** | **29336** | **28384** | **26866** | **5154** | **24246** | **18938** | **18044** | **18168** | **15528** | **9475** | 374641 |

**Table-B: Numbers of TBI YLLs in 16 European countries in 2013 by age group, for males**

| Country/Age group | 0-4 | 5-9 | 10-14 | 15-19 | 20-24 | 25-29 | 30-34 | 35-39 | 40-44 | 45-49 | 50-54 | 55-59 | 60-64 | 65-69 | 70-74 | 75-79 | 80-84 | 85 + | Total |
|---|---|---|---|---|---|---|---|---|---|---|---|---|---|---|---|---|---|---|---|
| **Austria** | 149 | 193 | 729 | 1016 | 765 | 809 | 581 | 785 | 1209 | 1430 | 1292 | 143 | 1139 | 884 | 1306 | 766 | 782 | 387 | **14365** |
| **Bulgaria** | 530 | 328 | 790 | 1756 | 921 | 1201 | 1412 | 1291 | 1147 | 1095 | 1086 | 211 | 1247 | 807 | 388 | 414 | 219 | 84 | **14927** |
| **Croatia** | 226 | 130 | 969 | 559 | 828 | 645 | 543 | 781 | 777 | 781 | 1162 | 0 | 834 | 433 | 403 | 481 | 268 | 115 | **9935** |
| **Cyprus** | 0 | 0 | 419 | 450 | 209 | 137 | 41 | 115 | 67 | 53 | 25 | 0 | 141 | 64 | 40 | 20 | 23 | 7 | **1811** |
| **Denmark** | 76 | 195 | 607 | 564 | 367 | 418 | 376 | 554 | 623 | 559 | 362 | 0 | 297 | 382 | 264 | 137 | 149 | 64 | **5994** |
| **Estonia** | 77 | 65 | 301 | 561 | 410 | 654 | 337 | 517 | 585 | 763 | 537 | 0 | 384 | 285 | 128 | 132 | 16 | 14 | **5766** |
| **Hungary** | 311 | 133 | 974 | 1348 | 1652 | 918 | 1413 | 1328 | 1478 | 1617 | 2247 | 0 | 1622 | 1204 | 966 | 550 | 435 | 208 | **18404** |
| **Ireland** | 77 | 128 | 303 | 851 | 574 | 697 | 252 | 450 | 395 | 338 | 462 | 285 | 263 | 244 | 103 | 80 | 56 | 38 | **5596** |
| **Italy** | 689 | 922 | 5395 | 7706 | 7002 | 5798 | 5101 | 5185 | 4978 | 4366 | 3030 | 423 | 3198 | 3209 | 2978 | 3691 | 2754 | 1383 | **67808** |
| **Lithuania** | 0 | 66 | 542 | 1015 | 828 | 981 | 1085 | 1227 | 1282 | 1680 | 1156 | 70 | 788 | 564 | 515 | 324 | 116 | 38 | **12277** |
| **Luxembourg** | 0 | 65 | 61 | 55 | 102 | 95 | 42 | 151 | 126 | 57 | 94 | 0 | 22 | 49 | 40 | 41 | 13 | 16 | **1029** |
| **Romania** | 1293 | 655 | 2788 | 3652 | 3251 | 2467 | 4051 | 4585 | 4317 | 3147 | 4063 | 502 | 3517 | 2015 | 1553 | 1041 | 542 | 217 | **43656** |
| **Serbia** | 227 | 261 | 1025 | 1789 | 1642 | 1965 | 1008 | 1152 | 1517 | 1157 | 1404 | 140 | 1682 | 777 | 591 | 817 | 395 | 73 | **17622** |
| **Slovakia** | 78 | 128 | 425 | 1027 | 878 | 1116 | 967 | 922 | 1386 | 1435 | 1524 | 217 | 1586 | 757 | 705 | 460 | 201 | 116 | **13928** |
| **Slovenia** | 0 | 0 | 0 | 332 | 102 | 284 | 126 | 184 | 69 | 256 | 356 | 0 | 280 | 133 | 184 | 179 | 121 | 60 | **2666** |
| **United Kingdom** | 986 | 398 | 3012 | 4618 | 4092 | 3118 | 3048 | 3550 | 4180 | 4280 | 2792 | 567 | 2370 | 2471 | 1717 | 2093 | 2363 | 1433 | **47088** |
| **Total** | **4719** | **3667** | **18340** | **27299** | **23623** | **21303** | **20383** | **22777** | **24136** | **23014** | **21592** | **2558** | **19370** | **14278** | **11881** | **11226** | **8453** | **4253** | **282872** |

**Table-C: Numbers of TBI YLLs in 16 European countries in 2013 by age group, for females**

| Country/Age group | 0-4 | 5-9 | 10-14 | 15-19 | 20-24 | 25-29 | 30-34 | 35-39 | 40-44 | 45-49 | 50-54 | 55-59 | 60-64 | 65-69 | 70-74 | 75-79 | 80-84 | 85 + | Total |
|---|---|---|---|---|---|---|---|---|---|---|---|---|---|---|---|---|---|---|---|
| **Austria** | 164 | 139 | 136 | 186 | 227 | 306 | 95 | 168 | 481 | 296 | 143 | 0 | 168 | 230 | 545 | 437 | 426 | 489 | **4636** |
| **Bulgaria** | 330 | 74 | 136 | 374 | 282 | 209 | 94 | 258 | 110 | 196 | 200 | 0 | 290 | 238 | 207 | 147 | 187 | 41 | **3373** |
| **Croatia** | 81 | 0 | 271 | 248 | 0 | 53 | 185 | 43 | 39 | 95 | 176 | 150 | 261 | 39 | 245 | 264 | 240 | 155 | **2545** |
| **Cyprus** | 0 | 0 | 0 | 0 | 0 | 0 | 45 | 0 | 36 | 0 | 0 | 0 | 47 | 18 | 14 | 23 | 19 | 11 | **213** |
| **Denmark** | 167 | 70 | 400 | 311 | 0 | 103 | 92 | 171 | 38 | 195 | 57 | 228 | 120 | 99 | 79 | 157 | 124 | 87 | **2498** |
| **Estonia** | 246 | 74 | 67 | 63 | 0 | 0 | 96 | 82 | 79 | 33 | 28 | 76 | 118 | 20 | 95 | 62 | 9 | 16 | **1164** |
| **Hungary** | 166 | 74 | 202 | 371 | 225 | 467 | 327 | 292 | 410 | 358 | 649 | 151 | 574 | 218 | 496 | 488 | 402 | 271 | **6141** |
| **Ireland** | 83 | 0 | 65 | 246 | 169 | 0 | 95 | 83 | 37 | 195 | 87 | 75 | 45 | 103 | 111 | 103 | 127 | 75 | **1699** |
| **Italy** | 1067 | 357 | 1596 | 2033 | 1311 | 1441 | 1228 | 1424 | 1280 | 1024 | 1080 | 306 | 1041 | 1041 | 1548 | 2229 | 2579 | 1897 | **24482** |
| **Lithuania** | 83 | 0 | 66 | 124 | 172 | 103 | 92 | 377 | 258 | 263 | 341 | 153 | 261 | 174 | 75 | 192 | 109 | 84 | **2927** |
| **Luxembourg** | 0 | 0 | 0 | 0 | 112 | 0 | 0 | 0 | 37 | 0 | 56 | 0 | 0 | 0 | 14 | 26 | 18 | 11 | **274** |
| **Romania** | 1147 | 570 | 1131 | 686 | 509 | 615 | 430 | 542 | 680 | 612 | 700 | 616 | 745 | 610 | 748 | 464 | 335 | 116 | **11256** |
| **Serbia** | 83 | 283 | 261 | 622 | 401 | 259 | 188 | 425 | 262 | 359 | 196 | 381 | 410 | 216 | 282 | 376 | 220 | 54 | **5278** |
| **Slovakia** | 0 | 214 | 266 | 183 | 112 | 159 | 49 | 296 | 327 | 129 | 312 | 154 | 117 | 217 | 203 | 227 | 280 | 114 | **3359** |
| **Slovenia** | 0 | 0 | 0 | 64 | 0 | 54 | 0 | 82 | 39 | 0 | 58 | 0 | 49 | 18 | 95 | 67 | 114 | 75 | **715** |
| **United Kingdom** | 1069 | 0 | 1535 | 1736 | 906 | 835 | 839 | 1343 | 1087 | 1615 | 1191 | 306 | 630 | 1419 | 1406 | 1680 | 1886 | 1726 | **21209** |
| **Total** | **4686** | **1855** | **6132** | **7247** | **4426** | **4604** | **3855** | **5586** | **5200** | **5370** | **5274** | **2596** | **4876** | **4660** | **6163** | **6942** | **7075** | **5222** | **91769** |

**Table-D: Crude TBI YLL rates per 100,000 in 16 European countries in 2013 by age group, for both sexes combined**

| Country/Age group | 0-4 | 5-9 | 10-14 | 15-19 | 20-24 | 25-29 | 30-34 | 35-39 | 40-44 | 45-49 | 50-54 | 55-59 | 60-64 | 65-69 | 70-74 | 75-79 | 80-84 | 85 + | Total |
|---|---|---|---|---|---|---|---|---|---|---|---|---|---|---|---|---|---|---|---|
| **Austria** | 78.6 | 82.2 | 207.5 | 253.0 | 183.5 | 200.5 | 119.9 | 174.2 | 263.4 | 242.5 | 216.8 | 26.1 | 277.2 | 267.5 | 422.9 | 456.4 | 554.3 | 424.6 | **224.1** |
| **Bulgaria** | 249.7 | 121.4 | 291.7 | 650.9 | 270.1 | 288.5 | 299.5 | 278.9 | 237.5 | 269.2 | 256.7 | 41.0 | 294.8 | 230.5 | 172.7 | 192.4 | 204.8 | 105.0 | **251.9** |
| **Croatia** | 145.1 | 63.0 | 579.2 | 328.0 | 328.9 | 251.3 | 247.1 | 287.9 | 292.9 | 292.2 | 429.0 | 47.5 | 388.1 | 224.5 | 317.3 | 419.9 | 431.2 | 396.1 | **293.3** |
| **Cyprus** | 0.0 | 0.0 | 914.8 | 793.5 | 301.7 | 185.5 | 120.9 | 185.8 | 173.1 | 92.8 | 43.5 | 0.0 | 399.2 | 215.1 | 179.1 | 191.5 | 281.6 | 162.5 | **234.8** |
| **Denmark** | 78.8 | 80.2 | 301.1 | 244.8 | 100.8 | 158.9 | 144.0 | 196.5 | 170.4 | 177.5 | 110.6 | 64.5 | 122.7 | 135.8 | 137.9 | 166.5 | 230.2 | 131.1 | **151.2** |
| **Estonia** | 431.2 | 193.9 | 605.0 | 995.8 | 461.1 | 673.4 | 474.8 | 661.1 | 723.6 | 936.3 | 610.5 | 85.7 | 610.9 | 488.0 | 355.6 | 378.3 | 67.6 | 113.3 | **525.8** |
| **Hungary** | 103.9 | 42.4 | 244.5 | 309.2 | 298.5 | 227.1 | 249.3 | 190.3 | 261.0 | 309.4 | 472.0 | 19.9 | 327.2 | 264.9 | 330.7 | 315.4 | 353.8 | 278.2 | **248.1** |
| **Ireland** | 43.5 | 38.6 | 119.0 | 396.8 | 290.9 | 218.4 | 90.3 | 146.1 | 125.6 | 170.5 | 192.7 | 142.7 | 137.4 | 181.8 | 153.6 | 172.7 | 250.8 | 181.0 | **158.6** |
| **Italy** | 64.3 | 45.0 | 247.3 | 342.5 | 268.1 | 220.9 | 171.4 | 148.5 | 129.7 | 109.5 | 93.8 | 18.8 | 116.5 | 126.6 | 148.1 | 227.9 | 267.9 | 179.6 | **153.2** |
| **Lithuania** | 54.9 | 48.9 | 412.8 | 624.3 | 467.4 | 557.2 | 669.1 | 859.7 | 735.0 | 905.0 | 632.9 | 113.6 | 611.9 | 539.0 | 417.5 | 431.7 | 262.1 | 207.4 | **514.1** |
| **Luxembourg** | 0.0 | 215.5 | 196.4 | 170.3 | 639.7 | 248.3 | 100.9 | 365.2 | 376.3 | 128.6 | 378.0 | 0.0 | 80.0 | 223.2 | 298.2 | 452.9 | 258.6 | 288.0 | **239.8** |
| **Romania** | 248.5 | 115.3 | 365.3 | 398.2 | 304.9 | 225.2 | 314.5 | 325.2 | 311.3 | 284.1 | 394.1 | 77.5 | 323.6 | 282.4 | 275.8 | 204.1 | 181.8 | 113.3 | **274.8** |
| **Serbia** | 93.5 | 157.5 | 366.3 | 620.8 | 477.1 | 469.5 | 242.5 | 313.5 | 373.8 | 319.6 | 314.5 | 93.2 | 376.7 | 264.3 | 262.6 | 409.6 | 332.7 | 138.0 | **319.6** |
| **Slovakia** | 26.8 | 126.0 | 258.2 | 391.0 | 257.9 | 300.1 | 226.1 | 268.7 | 452.9 | 430.6 | 496.0 | 95.8 | 498.2 | 404.6 | 494.3 | 512.0 | 496.3 | 347.6 | **319.3** |
| **Slovenia** | 0.0 | 0.0 | 0.0 | 405.6 | 89.0 | 245.2 | 81.5 | 171.8 | 73.6 | 163.9 | 271.2 | 0.0 | 240.4 | 154.9 | 307.8 | 334.8 | 430.6 | 337.9 | **164.1** |
| **United Kingdom** | 51.2 | 10.6 | 128.4 | 163.5 | 115.8 | 90.8 | 90.1 | 122.6 | 117.4 | 125.9 | 91.7 | 23.2 | 84.4 | 112.0 | 122.6 | 180.3 | 274.1 | 215.6 | **106.5** |

**Table-E: Crude TBI YLL rates per 100,000 in 16 European countries in 2013 by age group, for males**

| Country/Age group | 0-4 | 5-9 | 10-14 | 15-19 | 20-24 | 25-29 | 30-34 | 35-39 | 40-44 | 45-49 | 50-54 | 55-59 | 60-64 | 65-69 | 70-74 | 75-79 | 80-84 | 85 + | Total |
|---|---|---|---|---|---|---|---|---|---|---|---|---|---|---|---|---|---|---|---|
| **Austria** | 72.7 | 93.3 | 341.1 | 416.5 | 278.1 | 288.6 | 205.0 | 288.0 | 378.6 | 398.6 | 389.2 | 53.1 | 501.1 | 450.9 | 651.9 | 676.3 | 924.2 | 665.1 | **347.0** |
| **Bulgaria** | 299.6 | 192.5 | 484.2 | 1042.0 | 401.7 | 474.7 | 539.9 | 451.0 | 420.6 | 449.0 | 434.2 | 84.9 | 518.4 | 405.3 | 271.5 | 363.1 | 296.4 | 208.4 | **422.3** |
| **Croatia** | 207.6 | 122.4 | 883.3 | 444.4 | 643.1 | 456.6 | 361.3 | 538.5 | 555.1 | 524.4 | 757.8 | 0.0 | 620.9 | 462.1 | 468.8 | 704.3 | 674.3 | 634.1 | **483.9** |
| **Cyprus** | 0.0 | 0.0 | 1782.2 | 1547.5 | 591.6 | 374.8 | 120.7 | 405.2 | 246.3 | 196.6 | 89.3 | 0.0 | 608.1 | 348.5 | 279.3 | 196.6 | 366.7 | 160.0 | **431.9** |
| **Denmark** | 48.0 | 115.3 | 354.9 | 307.5 | 198.0 | 251.8 | 230.4 | 300.0 | 319.1 | 260.1 | 189.9 | 0.0 | 176.8 | 219.3 | 222.4 | 171.2 | 302.4 | 173.4 | **215.2** |
| **Estonia** | 200.4 | 176.1 | 962.0 | 1743.6 | 891.5 | 1306.2 | 720.1 | 1118.5 | 1268.4 | 1833.1 | 1217.6 | 0.0 | 1089.9 | 1130.1 | 559.7 | 802.5 | 156.5 | 258.0 | **936.7** |
| **Hungary** | 131.9 | 53.1 | 394.2 | 472.6 | 511.4 | 294.3 | 400.4 | 308.0 | 405.2 | 509.2 | 758.4 | 0.0 | 538.6 | 524.9 | 557.1 | 481.0 | 579.7 | 458.2 | **390.8** |
| **Ireland** | 41.1 | 75.5 | 191.8 | 597.7 | 442.3 | 456.3 | 135.9 | 249.4 | 229.6 | 216.7 | 326.7 | 227.2 | 235.7 | 256.0 | 151.7 | 163.5 | 181.3 | 188.1 | **245.9** |
| **Italy** | 49.1 | 63.1 | 371.0 | 525.6 | 441.6 | 351.0 | 275.9 | 233.1 | 207.3 | 179.2 | 141.2 | 22.5 | 182.4 | 200.8 | 211.8 | 326.7 | 353.0 | 246.4 | **232.3** |
| **Lithuania** | 0.0 | 95.6 | 715.1 | 1084.4 | 752.4 | 986.0 | 1225.8 | 1344.9 | 1268.1 | 1639.9 | 1047.0 | 79.2 | 1086.7 | 1050.9 | 1004.6 | 829.8 | 463.0 | 283.4 | **901.1** |
| **Luxembourg** | 0.0 | 419.9 | 382.6 | 332.2 | 594.3 | 489.2 | 202.4 | 726.9 | 569.6 | 247.4 | 460.4 | 0.0 | 157.7 | 449.8 | 477.2 | 633.6 | 268.8 | 584.4 | **378.6** |
| **Romania** | 256.2 | 120.0 | 506.2 | 654.6 | 507.5 | 347.8 | 555.9 | 569.8 | 525.6 | 466.2 | 675.9 | 72.8 | 579.9 | 489.4 | 450.4 | 364.7 | 305.4 | 220.5 | **447.6** |
| **Serbia** | 132.9 | 146.9 | 567.6 | 894.1 | 748.8 | 812.1 | 401.8 | 453.2 | 637.9 | 494.6 | 565.1 | 51.7 | 639.4 | 453.4 | 408.5 | 681.6 | 553.6 | 229.7 | **505.2** |
| **Slovakia** | 52.3 | 91.8 | 309.6 | 647.3 | 448.4 | 515.1 | 418.9 | 396.2 | 722.4 | 788.9 | 830.7 | 116.3 | 999.3 | 726.8 | 968.5 | 957.6 | 647.5 | 630.7 | **528.1** |
| **Slovenia** | 0.0 | 0.0 | 0.0 | 662.0 | 172.8 | 395.7 | 155.0 | 227.1 | 90.6 | 322.7 | 458.1 | 0.0 | 410.1 | 289.5 | 456.8 | 607.2 | 654.8 | 616.9 | **261.4** |
| **United Kingdom** | 48.0 | 20.7 | 166.1 | 231.5 | 186.9 | 143.5 | 142.3 | 178.7 | 188.5 | 185.3 | 129.9 | 30.5 | 136.1 | 146.4 | 142.4 | 219.3 | 362.9 | 290.5 | **149.3** |

**Table-F: Crude TBI YLL rates per 100,000 in 16 European countries in 2013 by age group, for females**

| Country/Age group | 0-4 | 5-9 | 10-14 | 15-19 | 20-24 | 25-29 | 30-34 | 35-39 | 40-44 | 45-49 | 50-54 | 55-59 | 60-64 | 65-69 | 70-74 | 75-79 | 80-84 | 85 + | Total |
|---|---|---|---|---|---|---|---|---|---|---|---|---|---|---|---|---|---|---|---|
| **Austria** | 84.9 | 70.5 | 67.0 | 80.4 | 85.5 | 110.9 | 33.9 | 61.2 | 149.3 | 83.8 | 43.3 | 0.0 | 68.8 | 104.3 | 229.6 | 290.8 | 319.5 | 330.1 | **106.8** |
| **Bulgaria** | 196.9 | 46.0 | 88.1 | 235.6 | 130.5 | 88.6 | 39.0 | 95.9 | 42.9 | 83.2 | 79.7 | 0.0 | 103.3 | 93.6 | 102.7 | 82.8 | 150.4 | 52.1 | **90.4** |
| **Croatia** | 78.9 | 0.0 | 259.6 | 206.2 | 0.0 | 38.8 | 128.2 | 30.5 | 28.1 | 63.0 | 111.0 | 93.2 | 176.6 | 33.5 | 207.2 | 241.9 | 307.5 | 309.9 | **115.6** |
| **Cyprus** | 0.0 | 0.0 | 0.0 | 0.0 | 0.0 | 0.0 | 121.0 | 0.0 | 111.5 | 0.0 | 0.0 | 0.0 | 196.6 | 91.1 | 88.5 | 187.2 | 219.8 | 164.2 | **48.1** |
| **Denmark** | 111.2 | 43.4 | 244.8 | 178.7 | 0.0 | 63.6 | 56.9 | 92.8 | 19.7 | 92.9 | 30.3 | 128.8 | 69.9 | 55.0 | 60.8 | 162.5 | 178.9 | 111.1 | **88.3** |
| **Estonia** | 674.5 | 212.8 | 226.8 | 206.6 | 0.0 | 0.0 | 216.3 | 184.8 | 173.1 | 76.1 | 57.8 | 157.4 | 251.4 | 53.6 | 238.5 | 178.0 | 33.7 | 76.0 | **165.7** |
| **Hungary** | 74.3 | 31.2 | 86.4 | 137.1 | 73.6 | 156.7 | 94.8 | 69.5 | 114.3 | 111.6 | 204.6 | 37.4 | 155.1 | 70.9 | 184.6 | 227.3 | 248.8 | 213.7 | **118.5** |
| **Ireland** | 46.0 | 0.0 | 43.0 | 183.5 | 134.5 | 0.0 | 47.8 | 45.0 | 21.5 | 124.5 | 60.6 | 59.2 | 40.0 | 107.8 | 155.4 | 180.6 | 301.9 | 177.5 | **73.1** |
| **Italy** | 80.4 | 25.9 | 116.3 | 147.6 | 86.5 | 88.6 | 66.6 | 63.9 | 52.8 | 41.2 | 48.3 | 15.3 | 55.2 | 59.2 | 93.8 | 151.9 | 213.0 | 149.9 | **78.9** |
| **Lithuania** | 112.8 | 0.0 | 92.3 | 139.6 | 165.5 | 108.4 | 105.3 | 395.4 | 237.9 | 234.3 | 270.4 | 141.8 | 263.9 | 209.0 | 83.3 | 238.5 | 179.3 | 184.9 | **183.5** |
| **Luxembourg** | 0.0 | 0.0 | 0.0 | 0.0 | 687.6 | 0.0 | 0.0 | 0.0 | 174.5 | 0.0 | 290.7 | 0.0 | 0.0 | 0.0 | 143.9 | 312.4 | 251.7 | 165.7 | **100.9** |
| **Romania** | 240.3 | 110.4 | 216.6 | 129.1 | 85.9 | 93.3 | 61.8 | 70.2 | 86.8 | 94.5 | 115.2 | 81.7 | 104.8 | 117.8 | 152.8 | 102.7 | 109.9 | 59.3 | **110.0** |
| **Serbia** | 51.7 | 168.6 | 153.1 | 330.4 | 191.9 | 111.8 | 77.6 | 170.9 | 110.1 | 149.4 | 75.3 | 132.1 | 140.3 | 105.7 | 150.2 | 219.4 | 193.9 | 89.6 | **143.6** |
| **Slovakia** | 0.0 | 162.2 | 204.0 | 121.4 | 59.6 | 76.4 | 22.4 | 134.1 | 175.5 | 71.1 | 167.1 | 76.8 | 63.9 | 158.9 | 183.0 | 263.5 | 425.0 | 238.6 | **121.0** |
| **Slovenia** | 0.0 | 0.0 | 0.0 | 134.8 | 0.0 | 81.7 | 0.0 | 111.1 | 55.2 | 0.0 | 77.4 | 0.0 | 71.5 | 34.9 | 188.7 | 152.3 | 315.8 | 248.1 | **68.8** |
| **United Kingdom** | 54.6 | 0.0 | 88.8 | 91.7 | 42.6 | 38.3 | 38.7 | 67.0 | 47.9 | 68.0 | 54.2 | 16.1 | 34.7 | 79.5 | 104.8 | 147.6 | 209.7 | 177.6 | **65.1** |
