# Supplementary figures and images for: Years of life lost due to traumatic brain injury in Europe: A cross-sectional analysis of 16 countries
Source: PLoS Med. 2017 Jul 11;14(7):e1002331. doi: 10.1371/journal.pmed.1002331 (PMC5507416; doi:10.1371/journal.pmed.1002331)

**S1 Figure: Age-standardized injury YLL rates and TBI YLL rates in 16 European countries by sex**

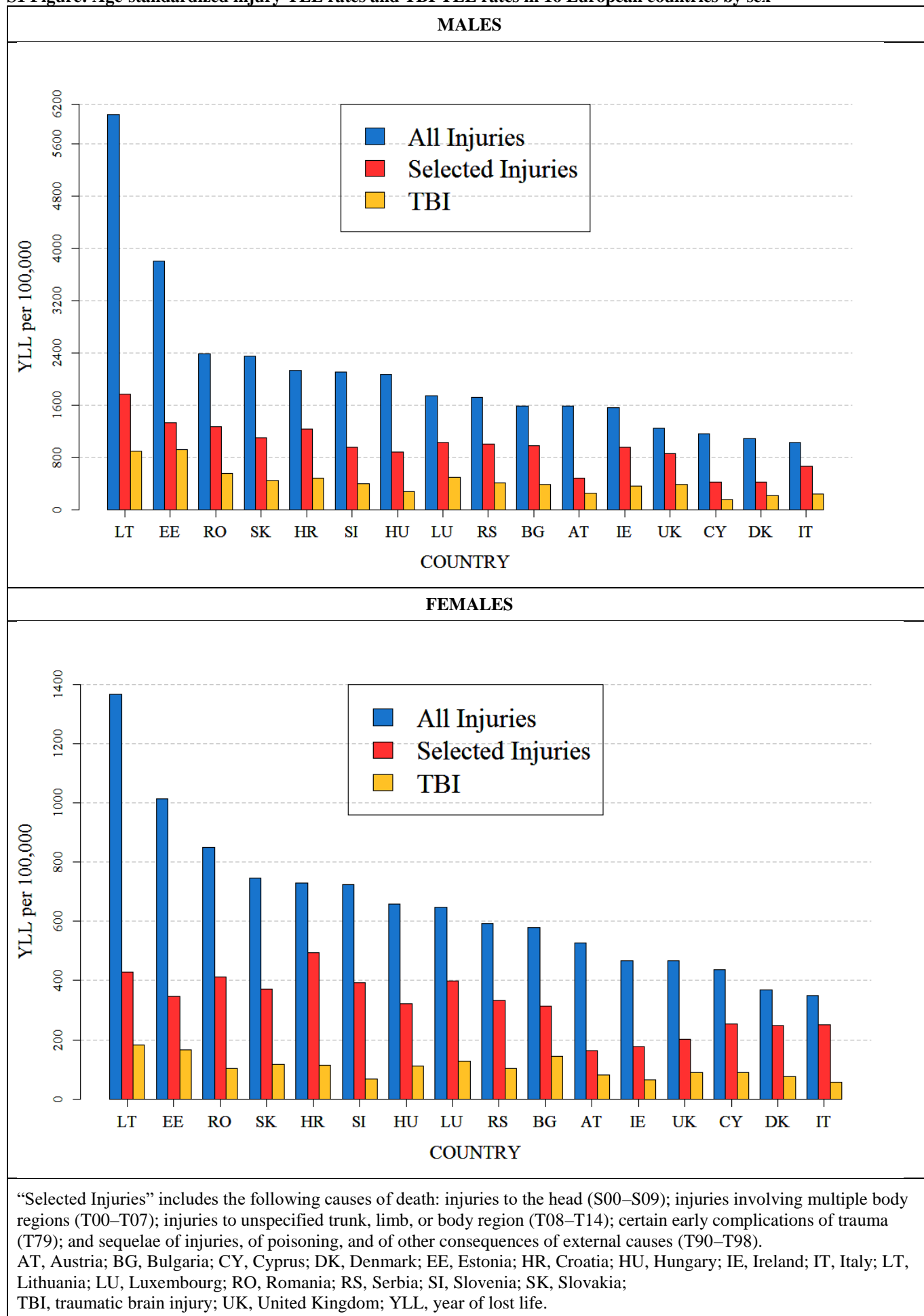

Supplement: S1 Fig — (PDF) [file pmed.1002331.s002.pdf]
