# Supplementary material for: Years of life lost due to traumatic brain injury in Europe: A cross-sectional analysis of 16 countries
Source: PLoS Med. 2017 Jul 11;14(7):e1002331. doi: 10.1371/journal.pmed.1002331 (PMC5507416; doi:10.1371/journal.pmed.1002331)
Supplement: S4 Table — (PDF) [file pmed.1002331.s007.pdf]

S4 Table. Comparison of pooled age-standardized rates of TBI YLLs per 100,000 persons in 16 European countries in 2013 calculated using the random effects model and the fixed effects model.

Model used	Both sexes	Males	Females
**Random effects model**	259.1 (205.8 to 312.3)	427.5 (290.0 to 564.9)	105.4 (89.1 to 121.6)
**Fixed effects model**	233,3 (233,1 to 233,5)	486.2 (485.6 to 486.9)	96.0 (95.9 to 96.2)
**I^2 with 95% CI**	100 % (100 % to 100 %)	100 % (100 % to 100 %)	100 % (100 % to 100 %)

TBI, traumatic brain injury; YLL, year of lost life.
