# Supplementary material for: Years of life lost due to traumatic brain injury in Europe: A cross-sectional analysis of 16 countries
Source: PLoS Med. 2017 Jul 11;14(7):e1002331. doi: 10.1371/journal.pmed.1002331 (PMC5507416; doi:10.1371/journal.pmed.1002331)
Supplement: S5 Table — (PDF) [file pmed.1002331.s008.pdf]

S5 Table: Numbers of TBI deaths in 16 European countries in 2013 by age-group, country and sex

	Age-group	0 - 4	5 - 14	15 - 34	35 - 64	65 - 84	85+	TOTAL
Total	Italy	22	29	594	1063	1819	1154	4681
	United Kingdom	26	18	361	881	1341	1131	3758
	Romania	31	33	276	923	577	87	1927
	Austria	4	7	77	274	471	282	1115
	Hungary	6	5	114	432	391	130	1078
	Serbia	4	15	149	342	313	36	859
	Slovakia	1	10	78	321	247	61	718
	Bulgaria	11	9	105	286	209	30	650
	Lithuania	1	4	72	294	165	29	565
	Croatia	4	4	65	202	209	67	551
	Denmark	3	7	49	113	114	58	344
	Ireland	2	7	54	90	74	35	262
	Estonia	4	3	39	121	55	8	230
	Slovenia	0	0	16	55	82	36	189
	Cyprus	0	0	22	20	18	7	67
	Luxembourg	0	1	8	19	17	10	55
	**Total**	**119**	**152**	**2079**	**5436**	**6102**	**3161**	**17049**
	**Proportion of total**	**1%**	**1%**	**12%**	**32%**	**36%**	**19%**	**100%**
Males	United Kingdom	13	14	278	684	818	534	2341
	Slovenia	0	0	14	48	56	13	131
	Slovakia	1	5	66	284	171	34	561
	Serbia	3	6	123	286	228	23	669
	Romania	17	17	227	808	421	56	1546
	Luxembourg	0	1	6	16	12	6	41
	Lithuania	0	2	64	245	123	11	445
	Italy	9	20	486	856	1179	533	3083
	Ireland	1	6	46	74	39	14	180
	Hungary	4	2	92	349	261	56	764
	Estonia	1	1	37	108	42	3	192
	Denmark	1	3	36	93	77	26	236
	Cyprus	0	0	22	16	12	5	55
	Croatia	3	2	56	176	142	31	410
	Bulgaria	7	8	88	250	150	22	525
	Austria	2	5	62	234	337	125	765
	**Total**	**62**	**92**	**1703**	**4527**	**4068**	**1492**	**11944**
	**Proportion of total**	**1%**	**1%**	**14%**	**38%**	**34%**	**12%**	**100%**
Females	Italy	13	9	108	207	640	621	1598
	United Kingdom	13	4	83	197	523	597	1417
	Romania	14	16	49	115	156	31	381
	Austria	2	2	15	40	134	157	350
	Hungary	2	3	22	83	130	74	314
	Serbia	1	9	26	56	85	13	190
	Slovakia	0	5	12	37	76	27	157
	Croatia	1	2	9	26	67	36	141
	Bulgaria	4	1	17	36	59	8	125
	Lithuania	1	2	8	49	42	18	120
	Denmark	2	4	13	20	37	32	108
	Ireland	1	1	8	16	35	21	82
	Slovenia	0	0	2	7	26	23	58
	Estonia	3	2	2	13	13	5	38
	Luxembourg	0	0	2	3	5	4	14
	Cyprus	0	0	0	4	6	2	12
	**Total**	**57**	**60**	**376**	**909**	**2034**	**1669**	**5105**
	**Proportion of total**	**1%**	**1%**	**7%**	**18%**	**40%**	**33%**	**100%**

TBI=Traumatic Brain Injury
