# Supplementary material for: Years of life lost due to traumatic brain injury in Europe: A cross-sectional analysis of 16 countries
Source: PLoS Med. 2017 Jul 11;14(7):e1002331. doi: 10.1371/journal.pmed.1002331 (PMC5507416; doi:10.1371/journal.pmed.1002331)
Supplement: S6 Table — (PDF) [file pmed.1002331.s009.pdf]

S6 Table: Crude and age-standardized TBI death rates in 16 European countries in 2013 by age and sex

	Age-group	0 - 4	5 - 14	15 - 34	35 - 64	65 - 84	85+	crude rate	Age-adjusted rate (95% CI)
**Total**	**Lithuania**	0.7	1.4	9.4	24.2	34.1	49.3	19.1	19.3 (17.7 - 20.9)
	**Estonia**	5.3	2.3	11.5	22.8	25.8	30.2	17.5	17.6 (15.4 - 20.1)
	**Slovakia**	0.3	1.9	5.0	14.0	37.7	92.2	13.3	15.8 (14.6 - 17.1)
	**Croatia**	1.9	1.0	6.1	11.4	29.4	98.3	13.0	13.7 (12.6 - 15)
	**Austria**	1.0	0.9	3.6	7.6	35.3	136.7	13.1	13.5 (12.7 - 14.3)
	**Serbia**	1.2	2.2	8.4	11.1	26.4	39.1	12.0	12.3 (11.5 - 13.2)
	**Luxembourg**	0.0	1.6	5.5	8.3	25.4	106.7	10.1	11.8 (8.9 - 15.5)
	**Hungary**	1.3	0.5	4.6	10.2	25.3	75.5	10.9	11.5 (10.8 - 12.2)
	**Romania**	3.2	1.5	5.4	10.9	19.3	29.6	9.6	10.1 (9.6 - 10.5)
	**Slovenia**	0.0	0.0	3.2	6.1	25.9	90.1	9.2	9.9 (8.5 - 11.4)
	**Cyprus**	3.2	1.4	6.0	9.2	16.2	25.2	8.9	8.9 (8.2 - 9.6)
	**Bulgaria**	0.0	0.0	8.1	6.0	17.0	63.2	7.8	8.9 (6.8 - 11.5)
	**Ireland**	0.8	0.5	4.6	4.1	16.5	63.2	7.8	7.2 (7 - 7.5)
	**Italy**	0.5	1.1	4.4	5.0	14.5	56.0	5.7	7.1 (6.2 - 8.1)
	**Denmark**	1.0	1.1	3.6	5.0	12.7	50.4	6.1	6.5 (5.8 - 7.2)
	**United Kingdom**	0.6	0.2	2.1	3.5	13.9	77.2	5.9	6.3 (6.1 - 6.5)
	**Pooled**								**11.3 (9.5 – 13.1)**
**Males**	**Lithuania**	0.0	1.4	16.3	43.3	72.8	82.0	32.7	36.0 (32.6 - 39.8)
	**Estonia**	2.6	1.5	21.1	42.6	56.2	55.3	31.2	33.5 (28.8 - 39.2)
	**Slovakia**	0.7	1.8	8.2	25.0	66.8	184.9	21.3	28.3 (25.7 - 31.2)
	**Croatia**	2.8	0.9	10.3	20.1	49.4	170.9	20.0	23.5 (21.1 - 26.2)
	**Austria**	1.0	1.2	5.7	13.2	56.7	214.8	18.5	21.9 (20.3 - 23.6)
	**Serbia**	1.8	1.7	13.5	19.0	44.9	72.4	19.2	20.6 (19.0 - 22.4)
	**Luxembourg**	0.0	3.2	8.1	13.6	39.2	219.1	15.1	20.0 (13.9 - 28.3)
	**Hungary**	1.7	0.4	7.2	16.9	44.1	123.3	16.2	19.3 (17.9 - 20.8)
	**Romania**	3.4	1.6	8.6	19.2	34.5	56.9	15.9	17.5 (16.6 - 18.4)
	**Slovenia**	0.0	0.0	5.3	10.4	41.7	133.7	12.8	15.9 (13.1 - 19.3)
	**Bulgaria**	4.0	2.4	9.6	16.2	28.3	54.6	14.9	15.5 (14.2 – 17.0)
	**Cyprus**	0.0	0.0	16.3	10.1	24.4	114.3	13.1	15.0 (11.0 - 20.3)
	**Italy**	0.6	0.7	7.4	6.7	24.0	95.0	10.6	11.0 (10.7 - 11.4)
	**Ireland**	0.5	1.8	7.5	8.3	16.0	69.3	7.9	9.9 (8.4 - 11.6)
	**Denmark**	0.6	0.9	5.2	8.2	18.2	70.5	8.5	9.6 (8.3 - 10.9)
	**United Kingdom**	0.6	0.4	3.3	5.6	18.2	108.3	7.4	8.9 (8.6 - 9.3)
	**Pooled**								**19.2 (15.4 - 22.9)**
**Females**	**Austria**	1.0	0.5	1.4	2.2	18.1	106.0	8.1	7.1 (6.3 - 7.9)
	**Lithuania**	1.4	1.5	2.1	7.6	13.4	39.6	7.5	7.1 (5.8 - 8.5)
	**Slovakia**	0.0	1.9	1.6	3.2	19.0	56.5	5.7	6.5 (5.5 - 7.7)
	**Croatia**	1.0	1.0	1.7	2.9	15.9	72.0	6.4	6.2 (5.2 - 7.4)
	**Hungary**	0.9	0.6	1.8	3.8	13.7	58.4	6.1	5.9 (5.2 - 6.6)
	**Luxembourg**	0.0	0.0	2.8	2.7	13.8	60.3	5.2	5.6 (3.0 - 9.5)
	**Slovenia**	0.0	0.0	0.8	1.6	14.3	76.1	5.6	5.2 (3.9 - 6.7)
	**Serbia**	0.6	2.7	3.0	3.6	12.6	21.6	5.2	5.2 (4.4 – 6.0)
	**Estonia**	8.2	3.1	1.2	4.7	9.4	23.8	5.4	5.1 (3.6 - 7.1)
	**Ireland**	0.6	0.3	1.3	1.8	13.2	49.7	3.5	4.6 (3.6 - 5.7)
	**United Kingdom**	0.7	0.1	1.0	1.6	10.1	61.4	4.3	4.2 (4.0 - 4.4)
	**Italy**	1.0	0.3	1.7	1.6	10.5	49.1	5.1	4.1 (3.9 - 4.4)
	**Romania**	2.9	1.5	2.0	2.7	8.8	15.9	3.7	3.8 (3.4 - 4.2)
	**Denmark**	1.3	1.2	1.9	1.8	7.8	40.9	3.8	3.7 (3.1 - 4.5)
	**Cyprus**	0.0	0.0	0.0	2.3	10.6	29.8	2.7	3.5 (1.8 - 6.2)
	**Bulgaria**	2.4	0.3	2.0	2.3	7.8	10.2	3.4	3.1 (2.6 - 3.8)
	**Pooled**								**5.1 (4.4 - 5.7)**

Meta-analysis heterogeneity: I^2 for total= 99.2% (95%CI: 99.1% to 99.4%); I^2 for males= 99.7% (95%CI: 99.7% to 99.7%); I^2 for females= 96.9% (95%CI: 96.0% to 97.6%);

TBI=Traumatic Brain Injury
