# Supplementary material for: Years of life lost due to traumatic brain injury in Europe: A cross-sectional analysis of 16 countries
Source: PLoS Med. 2017 Jul 11;14(7):e1002331. doi: 10.1371/journal.pmed.1002331 (PMC5507416; doi:10.1371/journal.pmed.1002331)
Supplement: S7 Table — (PDF) [file pmed.1002331.s010.pdf]

S7 Table: Male to female rate ratios of TBI death rates in 16 European countries in 2013

	RR
**Austria**	2.29 (2.02 to 2.60)
**Bulgaria**	4.43 (3.66 to 5.40)
**Croatia**	3.12 (2.58 to 3.78)
**Cyprus**	4.79 (2.65 to 9.41)
**Denmark**	2.22 (1.77 to 2.80)
**Estonia**	5.75 (4.11 to 8.26)
**Hungary**	2.68 (2.35 to 3.06)
**Ireland**	2.24 (1.73 to 2.92)
**Italy**	2.05 (1.93 to 2.18)
**Lithuania**	4.34 (3.56 to 5.33)
**Luxembourg**	2.90 (1.62 to 5.54)
**Romania**	4.25 (3.81 to 4.77)
**Serbia**	3.71 (3.16 to 4.37)
**Slovakia**	3.76 (3.16 to 4.50)
**Slovenia**	2.30 (1.70 to 3.15)
**United Kingdom**	1.71 (1.60 to 1.82)
**Total**	**2.46 (2.38 to 2.54)**

TBI=Traumatic Brain Injury
