# Supplementary material for: Years of life lost due to traumatic brain injury in Europe: A cross-sectional analysis of 16 countries
Source: PLoS Med. 2017 Jul 11;14(7):e1002331. doi: 10.1371/journal.pmed.1002331 (PMC5507416; doi:10.1371/journal.pmed.1002331)
Supplement: S8 Table — (PDF) [file pmed.1002331.s011.pdf]

**S8 Table. Rate ratios of TBI YLL rates per 100,000 persons in 16 European countries in 2013 by age and sex.**

| Age-group | 0 - 4 | 5 - 14 | 15 - 34 | 35 - 64 | 65 - 84 | 85+ |
|---|---|---|---|---|---|---|
| **Austria** | 0.36 (0.32 to 0.40) | 0.27 (0.24 to 0.29) | 0.90 (0.87 to 0.93) | 1 | 1.85 (1.79 to 1.92) | 1.95 (1.82 to 2.09) |
| **Bulgaria** | 0.92 (0.86 to 0.98) | 0.35 (0.32 to 0.38) | 1.18 (1.14 to 1.22) | 1 | 0.75 (0.71 to 0.78) | 0.39 (0.32 to 0.46) |
| **Croatia** | 0.45 (0.40 to 0.51) | 0.21 (0.18 to 0.23) | 1.04 (1.00 to 1.09) | 1 | 1.04 (1.00 to 1.10) | 1.24 (1.09 to 1.40) |
| **Cyprus** | - | - | 2.62 (2.38 to 2.90) | 1 | 1.21 (1.04 to 1.42) | 0.96 (0.58 to 1.49) |
| **Denmark** | 0.52 (0.45 to 0.59) | 0.49 (0.44 to 0.53) | 1.32 (1.26 to 1.39) | 1 | 1.01 (0.95 to 1.08) | 0.86 (0.73 to 1.01) |
| **Estonia** | 0.64 (0.57 to 0.72) | 0.24 (0.21 to 0.28) | 0.90 (0.85 to 0.95) | 1 | 0.52 (0.48 to 0.56) | 0.17 (0.12 to 0.24) |
| **Hungary** | 0.36 (0.33 to 0.39) | 0.13 (0.11 to 0.14) | 0.85 (0.83 to 0.88) | 1 | 1.06 (1.03 to 1.10) | 0.96 (0.88 to 1.05) |
| **Ireland** | 0.29 (0.24 to 0.34) | 0.50 (0.46 to 0.55) | 1.55 (1.47 to 1.63) | 1 | 1.20 (1.11 to 1.29) | 1.20 (0.98 to 1.44) |
| **Italy** | 0.51 (0.49 to 0.53) | 0.28 (0.27 to 0.29) | 1.98 (1.95 to 2.01) | 1 | 1.44 (1.42 to 1.47) | 1.42 (1.37 to 1.47) |
| **Lithuania** | 0.08 (0.06 to 0.09) | 0.14 (0.13 to 0.16) | 0.69 (0.66 to 0.72) | 1 | 0.59 (0.56 to 0.62) | 0.29 (0.24 to 0.34) |
| **Luxembourg** | - | 0.42 (0.32 to 0.54) | 1.15 (1.01 to 1.30) | 1 | 1.19 (1.01 to 1.39) | 1.14 (0.75 to 1.64) |
| **Romania** | 0.77 (0.74 to 0.80) | 0.34 (0.33 to 0.35) | 0.91 (0.90 to 0.93) | 1 | 0.76 (0.74 to 0.78) | 0.35 (0.31 to 0.39) |
| **Serbia** | 0.30 (0.26 to 0.33) | 0.48 (0.45 to 0.51) | 1.41 (1.37 to 1.45) | 1 | 0.98 (0.94 to 1.02) | 0.43 (0.36 to 0.52) |
| **Slovakia** | 0.07 (0.05 to 0.08) | 0.34 (0.31 to 0.36) | 0.67 (0.65 to 0.70) | 1 | 1.18 (1.13 to 1.23) | 0.88 (0.77 to 1.00) |
| **Slovenia** | - | - | 0.99 (0.91 to 1.08) | 1 | 1.73 (1.59 to 1.87) | 2.03 (1.69 to 2.41) |
| **United Kingdom** | 0.47 (0.45 to 0.49) | 0.16 (0.15 to 0.17) | 1.08 (1.06 to 1.10) | 1 | 1.43 (1.41 to 1.46) | 1.99 (1.92 to 2.06) |
| **Total** | **0.45 (0.45 to 0.46)** | **0.26 (0.26 to 0.27)** | **1.18 (1.17 to 1.19)** | **1** | **1.13 (1.12 to 1.14)** | **1.07 (1.05 to 1.10)** |
| **Austria** | 0.20 (0.17 to 0.24) | 0.22 (0.20 to 0.25) | 0.85 (0.81 to 0.88) | 1 | 1.74 (1.67 to 1.81) | 1.84 (1.66 to 2.04) |
| **Bulgaria** | 0.63 (0.58 to 0.69) | 0.34 (0.31 to 0.37) | 1.08 (1.04 to 1.12) | 1 | 0.73 (0.69 to 0.77) | 0.44 (0.35 to 0.55) |
| **Croatia** | 0.37 (0.33 to 0.43) | 0.11 (0.09 to 0.13) | 0.99 (0.94 to 1.03) | 1 | 0.99 (0.93 to 1.05) | 1.14 (0.94 to 1.36) |
| **Cyprus** | - | - | 3.23 (2.90 to 3.61) | 1 | 1.07 (0.88 to 1.28) | 0.59 (0.25 to 1.15) |
| **Denmark** | 0.20 (0.16 to 0.24) | 0.23 (0.20 to 0.27) | 1.14 (1.08 to 1.21) | 1 | 0.90 (0.83 to 0.97) | 0.71 (0.55 to 0.90) |
| **Estonia** | 0.16 (0.13 to 0.20) | 0.08 (0.06 to 0.10) | 0.89 (0.84 to 0.95) | 1 | 0.61 (0.56 to 0.67) | 0.21 (0.12 to 0.34) |
| **Hungary** | 0.28 (0.25 to 0.31) | 0.06 (0.05 to 0.07) | 0.82 (0.79 to 0.85) | 1 | 1.13 (1.09 to 1.18) | 0.97 (0.85 to 1.12) |
| **Ireland** | 0.17 (0.13 to 0.21) | 0.52 (0.47 to 0.58) | 1.63 (1.54 to 1.73) | 1 | 0.81 (0.74 to 0.90) | 0.78 (0.55 to 1.05) |
| **Italy** | 0.24 (0.23 to 0.26) | 0.23 (0.22 to 0.24) | 1.96 (1.93 to 2.00) | 1 | 1.28 (1.25 to 1.30) | 1.22 (1.16 to 1.29) |
| **Lithuania** | - | 0.07 (0.06 to 0.09) | 0.67 (0.65 to 0.70) | 1 | 0.70 (0.67 to 0.74) | 0.22 (0.16 to 0.30) |
| **Luxembourg** | - | 0.49 (0.38 to 0.64) | 1.01 (0.88 to 1.17) | 1 | 1.12 (0.93 to 1.35) | 1.41 (0.82 to 2.24) |
| **Romania** | 0.45 (0.43 to 0.48) | 0.19 (0.18 to 0.20) | 0.82 (0.80 to 0.84) | 1 | 0.75 (0.73 to 0.77) | 0.39 (0.34 to 0.44) |
| **Serbia** | 0.25 (0.22 to 0.29) | 0.21 (0.19 to 0.24) | 1.34 (1.30 to 1.39) | 1 | 0.97 (0.93 to 1.01) | 0.44 (0.34 to 0.55) |
| **Slovakia** | 0.08 (0.06 to 0.09) | 0.18 (0.16 to 0.20) | 0.62 (0.60 to 0.65) | 1 | 1.20 (1.15 to 1.26) | 0.92 (0.76 to 1.10) |
| **Slovenia** | - | - | 0.99 (0.90 to 1.08) | 1 | 1.66 (1.51 to 1.83) | 2.24 (1.71 to 2.87) |
| **United Kingdom** | 0.29 (0.27 to 0.31) | 0.16 (0.15 to 0.17) | 1.06 (1.04 to 1.08) | 1 | 1.16 (1.14 to 1.19) | 1.76 (1.67 to 1.86) |
| **Total** | **0.27 (0.26 to 0.28)** | **0.18 (0.18 to 0.19)** | **1.13 (1.12 to 1.14)** | **1** | **1.00 (0.99 to 1.02)** | **0.93 (0.90 to 0.96)** |
| **Austria** | 1.13 (0.96 to 1.33) | 0.46 (0.39 to 0.55) | 1.09 (1.00 to 1.18) | 1 | 2.95 (2.75 to 3.17) | 4.41 (3.97 to 4.89) |
| **Bulgaria** | 2.67 (2.36 to 3.02) | 0.32 (0.25 to 0.40) | 1.59 (1.46 to 1.74) | 1 | 1.40 (1.27 to 1.53) | 0.71 (0.51 to 0.95) |
| **Croatia** | 0.89 (0.70 to 1.11) | 0.82 (0.69 to 0.98) | 1.23 (1.10 to 1.37) | 1 | 2.10 (1.90 to 2.32) | 3.48 (2.92 to 4.13) |
| **Cyprus** | - | - | - | 1 | 1.77 (1.32 to 2.35) | 2.27 (1.15 to 4.02) |
| **Denmark** | 1.86 (1.57 to 2.20) | 1.54 (1.34 to 1.76) | 2.01 (1.82 to 2.23) | 1 | 1.61 (1.43 to 1.82) | 1.86 (1.48 to 2.31) |
| **Estonia** | 4.29 (3.66 to 5.01) | 1.47 (1.22 to 1.77) | 0.50 (0.41 to 0.61) | 1 | 0.85 (0.72 to 1.01) | 0.49 (0.28 to 0.78) |
| **Hungary** | 0.62 (0.53 to 0.73) | 0.40 (0.35 to 0.46) | 0.87 (0.81 to 0.93) | 1 | 1.41 (1.33 to 1.50) | 1.79 (1.58 to 2.03) |
| **Ireland** | 0.76 (0.60 to 0.95) | 0.40 (0.31 to 0.50) | 1.26 (1.12 to 1.43) | 1 | 2.76 (2.43 to 3.13) | 2.94 (2.27 to 3.71) |
| **Italy** | 1.51 (1.41 to 1.61) | 0.45 (0.42 to 0.49) | 1.88 (1.82 to 1.94) | 1 | 2.28 (2.20 to 2.35) | 2.81 (2.67 to 2.95) |
| **Lithuania** | 0.46 (0.37 to 0.57) | 0.45 (0.38 to 0.53) | 0.51 (0.46 to 0.56) | 1 | 0.71 (0.65 to 0.78) | 0.76 (0.60 to 0.93) |
| **Luxembourg** | - | - | 1.88 (1.43 to 2.49) | 1 | 1.93 (1.39 to 2.68) | 2.03 (1.02 to 3.62) |
| **Romania** | 2.77 (2.59 to 2.96) | 1.32 (1.23 to 1.40) | 1.37 (1.30 to 1.44) | 1 | 1.41 (1.34 to 1.49) | 0.68 (0.57 to 0.82) |
| **Serbia** | 0.44 (0.35 to 0.55) | 1.67 (1.53 to 1.83) | 1.51 (1.41 to 1.61) | 1 | 1.38 (1.28 to 1.48) | 0.77 (0.58 to 0.99) |
| **Slovakia** | - | 1.32 (1.18 to 1.48) | 0.89 (0.81 to 0.97) | 1 | 2.19 (2.01 to 2.38) | 2.25 (1.85 to 2.71) |
| **Slovenia** | - | - | 0.94 (0.75 to 1.17) | 1 | 3.10 (2.61 to 3.69) | 4.77 (3.65 to 6.16) |
| **United Kingdom** | 1.02 (0.96 to 1.09) | 0.16 (0.14 to 0.18) | 1.12 (1.08 to 1.16) | 1 | 2.32 (2.24 to 2.40) | 3.33 (3.15 to 3.51) |
| **Total** | **1.26 (1.22 to 1.30)** | **0.61 (0.59 to 0.63)** | **1.29 (1.26 to 1.31)** | **1** | **1.92 (1.89 to 1.95)** | **2.34 (2.27 to 2.41)** |

The age group 35–64 years (rate ratio “1”) was used as the reference category when calculating rate ratios.

TBI, traumatic brain injury; YLL, year of lost life.
