# Supplementary material for: Years of life lost due to traumatic brain injury in Europe: A cross-sectional analysis of 16 countries
Source: PLoS Med. 2017 Jul 11;14(7):e1002331. doi: 10.1371/journal.pmed.1002331 (PMC5507416; doi:10.1371/journal.pmed.1002331)
Supplement: S9 Table — (PDF) [file pmed.1002331.s012.pdf]

S9 Table: Male to female rate ratios of TBI YLL rates in 2013 by country

	RR
**Austria**	3.25 (3.14 to 3.36)
**Bulgaria**	4.67 (4.50 to 4.85)
**Croatia**	4.19 (4.01 to 4.37)
**Cyprus**	9.01 (7.83 to 10.41)
**Denmark**	2.44 (2.33 to 2.56)
**Estonia**	5.66 (5.31 to 6.03)
**Hungary**	3.30 (3.21 to 3.40)
**Ireland**	3.36 (3.18 to 3.55)
**Italy**	2.95 (2.90 to 2.99)
**Lithuania**	4.91 (4.72 to 5.11)
**Luxembourg**	3.76 (3.30 to 4.30)
**Romania**	4.07 (3.98 to 4.15)
**Serbia**	3.52 (3.41 to 3.63)
**Slovakia**	4.36 (4.20 to 4.53)
**Slovenia**	3.79 (3.50 to 4.12)
**United Kingdom**	2.29 (2.26 to 2.33)
**Total**	**3.24 (3.22 to 3.27)**

TBI=Traumatic Brain Injury, YLL=Years of Lost Life
