# Supplementary material for: Years of life lost due to traumatic brain injury in Europe: A cross-sectional analysis of 16 countries
Source: PLoS Med. 2017 Jul 11;14(7):e1002331. doi: 10.1371/journal.pmed.1002331 (PMC5507416; doi:10.1371/journal.pmed.1002331)
Supplement: S10 Table — (PDF) [file pmed.1002331.s013.pdf]

S10 Table. Total numbers of injury YLLs in 16 European countries in 2013 by age group and sex.

	Age-group	0 - 4	5 - 14	15 - 34	35 - 64	65 - 84	85+	TOTAL
Total	Italy	3151	3913	83450	101959	63758	18850	275081
	United Kingdom	2835	2949	62746	70060	37546	10354	186490
	Romania	4299	6566	45434	69166	16678	697	142840
	Hungary	628	911	14694	31608	13866	2359	64066
	Austria	387	967	15463	23207	11768	2536	54328
	Serbia	475	1571	16490	22196	7457	381	48570
	Bulgaria	1340	1158	16088	21876	5561	393	46416
	Slovakia	155	1125	12576	20545	7401	1085	42887
	Croatia	542	644	11334	13487	8535	1649	36191
	Lithuania	238	650	8786	17614	3955	351	31594
	Denmark	321	558	4762	6632	3824	1044	17141
	Ireland	396	489	5319	5142	2149	315	13810
	Slovenia	0	279	3499	5082	3261	770	12891
	Estonia	323	291	3390	5420	1232	64	10720
	Cyprus	0	139	2151	1442	837	143	4712
	Luxembourg	77	130	981	1792	577	126	3683
	**Total**	**15167**	**22340**	**307163**	**417228**	**188405**	**41117**	**991420**
	**Proportion of total**	**2%**	**2%**	**31%**	**42%**	**19%**	**4%**	**100%**
Males	Italy	1518	2670	67204	78549	35233	6153	191327
	United Kingdom	1519	1914	48867	52870	19854	3821	128845
	Romania	2660	3743	35288	56938	10954	354	109937
	Hungary	462	612	11198	24096	7221	664	44253
	Austria	223	534	11685	18414	7705	828	39389
	Serbia	227	608	13378	17767	4882	141	37003
	Bulgaria	607	935	13442	17831	3802	204	36821
	Slovakia	155	609	10198	16762	4334	309	32367
	Croatia	378	338	9245	10521	4163	383	25028
	Lithuania	75	271	7620	14136	2424	106	24632
	Denmark	154	260	3465	5061	2153	350	11443
	Ireland	151	414	4413	4046	1281	95	10400
	Slovenia	0	203	2534	3900	1864	185	8686
	Estonia	77	65	2740	4609	773	34	8298
	Cyprus	0	139	1971	1095	503	41	3749
	Luxembourg	77	130	582	1391	363	36	2579
	**Total**	**8283**	**13445**	**243830**	**327986**	**107509**	**13704**	**714757**
	**Proportion of total**	**1%**	**2%**	**34%**	**46%**	**15%**	**2%**	**100%**
Females	Italy	1633	1243	16246	23410	28525	12697	83754
	United Kingdom	1316	1035	13879	17190	17692	6533	57645
	Romania	1639	2823	10146	12228	5724	343	32903
	Hungary	166	299	3496	7512	6645	1695	19813
	Austria	164	433	3778	4793	4063	1708	14939
	Serbia	248	963	3112	4429	2575	240	11567
	Croatia	164	306	2089	2966	4372	1266	11163
	Slovakia	0	516	2378	3783	3067	776	10520
	Bulgaria	733	223	2646	4045	1759	189	9595
	Lithuania	163	379	1166	3478	1531	245	6962
	Denmark	167	298	1297	1571	1671	694	5698
	Slovenia	0	76	965	1182	1397	585	4205
	Ireland	245	75	906	1096	868	220	3410
	Estonia	246	226	650	811	459	30	2422
	Luxembourg	0	0	399	401	214	90	1104
	Cyprus	0	0	180	347	334	102	963
	**Total**	**6884**	**8895**	**63333**	**89242**	**80896**	**27413**	**276663**
	**Proportion of total**	**2%**	**3%**	**23%**	**32%**	**29%**	**10%**	**100%**

Included causes of death: injuries to the head (S00–S09); injuries involving multiple body regions (T00–T07); injuries to unspecified trunk, limb, or body region (T08–T14); certain early complications of trauma (T79); and sequelae of injuries, of poisoning, and of other consequences of external causes (T90–T98). YLL, year of lost life.
