# Supplementary material for: Years of life lost due to traumatic brain injury in Europe: A cross-sectional analysis of 16 countries
Source: PLoS Med. 2017 Jul 11;14(7):e1002331. doi: 10.1371/journal.pmed.1002331 (PMC5507416; doi:10.1371/journal.pmed.1002331)
Supplement: S11 Table — (PDF) [file pmed.1002331.s014.pdf]

S11 Table. Crude and age-adjusted injury YLL rates in 16 European countries by age group and sex.

	Age-group	0 - 4	5 - 14	15 - 34	35 - 64	65 - 84	85+	crude rate	age-adjusted rate (95% CI)
**Total**	**Lithuania**	157.6	230.3	1145.7	1449.7	817.8	596.6	1068.2	1049.2 (1037.7 - 1060.9)
	**Croatia**	256.2	153.1	1059.1	760.1	1202.5	2419.4	850.7	856.6 (847.7 - 865.6)
	**Slovakia**	53.2	208.7	802.3	895.6	1129.0	1639.8	792.2	813.8 (805.9 - 821.8)
	**Estonia**	431.2	219.6	997.3	1021.2	577.2	241.7	813.4	802.8 (787.7 - 818.2)
	**Romania**	437.7	307.5	888.1	816.3	559.0	237.1	714.8	700.4 (696.8 - 704.1)
	**Luxembourg**	251.1	212.3	673.7	780.3	863.3	1343.9	677.8	694.0 (671.4 - 717.2)
	**Serbia**	143.3	225.5	924.7	721.5	629.7	414.0	678.0	663.5 (657.6 - 669.4)
	**Hungary**	136.7	94.0	589.6	742.7	897.7	1369.9	647.6	646.3 (641.3 - 651.4)
	**Slovenia**	0.0	147.8	693.2	565.0	1031.4	1927.2	625.8	635.0 (624.0 - 646.1)
	**Austria**	97.2	117.8	724.0	647.8	881.1	1229.1	640.7	634.4 (629.1 - 639.8)
	**Bulgaria**	389.0	178.5	911.9	705.4	431.9	330.1	638.9	625.3 (619.6 – 631.0)
	**Cyprus**	0.0	152.0	793.8	432.3	792.2	1291.2	546.7	549.3 (533.1 - 565.9)
	**Italy**	115.4	69.0	646.2	390.7	579.6	1032.0	456.7	451.0 (449.3 - 452.7)
	**Ireland**	107.7	76.3	430.6	288.4	422.1	504.4	300.3	318.3 (312.9 - 323.8)
	**Denmark**	104.1	83.9	346.5	294.2	425.8	906.3	305.3	311.8 (307.1 - 316.5)
	**United Kingdom**	70.7	40.4	371.9	282.3	388.6	706.6	290.8	295.3 (293.9 - 296.6)
	**Pooled**								**627.9 (522.9 – 733.0)**
**Males**	**Lithuania**	97.0	187.4	1945.5	2497.1	1433.8	786.8	1807.8	1767.1 (1744.7 - 1789.8)
	**Estonia**	200.4	95.4	1565.4	1816.9	1034.2	622.8	1348.1	1323.5 (1294.6 - 1353.1)
	**Slovakia**	103.7	220.0	1271.6	1476.7	1692.7	1680.7	1227.1	1262.4 (1247.7 - 1277.3)
	**Croatia**	347.0	156.3	1692.9	1200.4	1446.8	2111.7	1219.0	1225.8 (1210.2 - 1241.6)
	**Romania**	526.9	341.3	1338.4	1356.3	898.3	360.1	1127.1	1096.1 (1089.5 - 1102.7)
	**Serbia**	133.0	169.7	1466.5	1178.0	962.4	445.0	1060.7	1029.1 (1018.5 - 1039.8)
	**Bulgaria**	343.0	280.4	1473.3	1156.3	717.5	506.4	1041.6	1004.5 (994.2 – 1015.0)
	**Luxembourg**	488.7	414.3	787.8	1184.8	1185.3	1318.5	948.8	973.4 (934.9 - 1013.6)
	**Austria**	109.0	126.9	1079.3	1035.1	1296.5	1422.6	951.5	953.6 (944.1 - 963.2)
	**Hungary**	195.9	123.0	879.6	1166.6	1219.4	1462.6	939.6	950.7 (941.6 – 960.0)
	**Slovenia**	0.0	209.3	966.4	848.5	1389.1	1904.2	851.7	882.3 (863.2 - 901.9)
	**Cyprus**	0.0	296.0	1461.0	690.2	1023.2	939.6	894.1	860.2 (831.7 - 889.7)
	**Italy**	108.2	91.6	1025.6	611.8	716.9	1096.2	655.5	658.7 (655.7 - 661.6)
	**Ireland**	80.6	126.3	723.0	456.2	527.0	467.8	457.0	477.5 (468.0 - 487.2)
	**Denmark**	97.1	76.5	496.4	447.8	509.9	948.7	410.8	424.2 (416.4 - 432.2)
	**United Kingdom**	73.9	51.2	574.9	431.2	441.3	774.6	408.5	416.3 (414.0 - 418.6)
	**Pooled**								**956.6 (782.6 - 1130.6)**
**Females**	**Croatia**	159.6	149.3	398.7	330.3	1035.9	2531.0	507.1	493.2 (484.1 - 502.5)
	**Lithuania**	221.3	275.9	310.7	535.9	486.5	539.4	436.4	429.2 (419.1 - 439.5)
	**Luxembourg**	0.0	0.0	556.2	357.3	591.1	1355.9	406.6	412.6 (388.4 – 438.0)
	**Slovakia**	0.0	196.6	310.6	326.5	767.7	1623.8	379.0	399.5 (391.8 - 407.3)
	**Slovenia**	0.0	82.5	397.7	268.7	767.8	1935.2	404.3	392.3 (380.5 - 404.4)
	**Hungary**	74.2	63.4	286.7	343.0	697.7	1336.6	382.2	370.4 (365.3 - 375.7)
	**Estonia**	673.1	351.3	394.1	292.7	330.9	141.6	344.6	345.2 (331.5 - 359.3)
	**Austria**	84.8	108.2	358.7	265.8	548.1	1152.9	344.2	331.9 (326.5 - 337.3)
	**Romania**	343.3	271.8	409.2	286.0	324.5	175.2	321.6	320.7 (317.2 - 324.1)
	**Serbia**	154.3	284.5	357.2	282.4	380.3	397.7	314.6	313.7 (308.0 - 319.5)
	**Bulgaria**	437.5	70.8	310.6	259.4	232.2	239.5	257.2	254.7 (249.6 - 259.9)
	**Cyprus**	0.0	0.0	132.5	198.5	590.8	1525.1	217.7	250.8 (234.8 - 267.6)
	**Italy**	123.1	45.1	255.4	176.6	468.7	1003.6	269.8	248.7 (247.0 - 250.4)
	**Denmark**	111.0	91.7	191.8	139.8	351.0	886.0	201.3	200.1 (194.9 - 205.4)
	**United Kingdom**	67.2	29.1	165.8	136.9	342.6	672.2	176.9	176.8 (175.4 - 178.3)
	**Ireland**	135.8	23.8	145.0	122.4	326.1	521.2	146.8	162.3 (156.7 – 168.0)
	**Pooled**								**318.9 (271.1 - 366.6)**

Included causes of death: injuries to the head (S00–S09); injuries involving multiple body regions (T00–T07); injuries to unspecified trunk, limb, or body region (T08–T14); certain early complications of trauma (T79); and sequelae of injuries, of poisoning, and of other consequences of external causes (T90–T98). YLL, year of lost life.

Meta-analysis heterogeneity: I^2 for total= 99.9% (95%CI: 99.9% to 99.9%); I^2 for males= 99.9% (95%CI: 99.9% to 99.9%); I^2 for females= 99.7% (95%CI: 99.7% to 99.7%);
