# Supplementary material for: Years of life lost due to traumatic brain injury in Europe: A cross-sectional analysis of 16 countries
Source: PLoS Med. 2017 Jul 11;14(7):e1002331. doi: 10.1371/journal.pmed.1002331 (PMC5507416; doi:10.1371/journal.pmed.1002331)
Supplement: S12 Table — (PDF) [file pmed.1002331.s015.pdf]

S12 Table. Contribution of TBI YLLs to injury YLLs by country, age group, and sex.

Age-group	0 - 4	5 - 14	15 - 34	35 - 64	65 - 84	85+	TOTAL
**Estonia**	100%	74%	61%	66%	61%	47%	65%
**Ireland**	40%	100%	55%	53%	43%	36%	53%
**Denmark**	76%	88%	58%	52%	36%	14%	50%
**Lithuania**	35%	44%	44%	50%	52%	35%	48%
**Serbia**	65%	68%	48%	44%	49%	33%	47%
**Cyprus**	-	0	56%	40%	26%	13%	43%
**Slovakia**	50%	63%	33%	44%	41%	21%	40%
**Bulgaria**	64%	53%	35%	39%	47%	32%	39%
**Romania**	57%	36%	33%	40%	44%	48%	38%
**Hungary**	76%	39%	42%	39%	34%	20%	38%
**United Kingdom**	72%	43%	32%	38%	40%	31%	37%
**Luxembourg**	0	50%	43%	33%	35%	21%	35%
**Austria**	81%	49%	27%	34%	46%	35%	35%
**Croatia**	57%	43%	32%	42%	28%	16%	34%
**Italy**	56%	51%	39%	32%	31%	17%	34%
**Slovenia**	-	0	24%	30%	28%	18%	26%
**Average**	**59%**	**50%**	**41%**	**42%**	**40%**	**27%**	**41%**
**Estonia**	100%	100%	70%	68%	72%	42%	69%
**Ireland**	51%	100%	55%	53%	38%	40%	54%
**Denmark**	49%	75%	56%	55%	43%	18%	52%
**Lithuania**	0%	50%	44%	51%	63%	36%	50%
**Cyprus**	-	0%	62%	40%	29%	18%	48%
**Serbia**	100%	66%	48%	45%	53%	52%	48%
**Slovakia**	50%	57%	34%	47%	49%	37%	43%
**Hungary**	67%	22%	44%	40%	44%	31%	42%
**Bulgaria**	87%	58%	35%	41%	48%	41%	41%
**Luxembourg**	0%	50%	54%	35%	40%	43%	40%
**Romania**	49%	31%	34%	42%	47%	61%	40%
**Croatia**	60%	39%	32%	46%	38%	30%	40%
**United Kingdom**	65%	50%	30%	38%	44%	38%	37%
**Austria**	67%	63%	28%	35%	49%	47%	36%
**Italy**	45%	50%	39%	33%	36%	22%	35%
**Slovenia**	-	0%	28%	33%	33%	33%	31%
**Average**	**56%**	**51%**	**43%**	**44%**	**45%**	**37%**	**44%**
**Ireland**	34%	100%	53%	50%	51%	34%	50%
**Estonia**	100%	66%	20%	54%	40%	53%	48%
**Serbia**	33%	69%	50%	42%	43%	23%	46%
**Denmark**	100%	100%	63%	43%	27%	12%	44%
**Lithuania**	51%	40%	40%	46%	36%	34%	42%
**United Kingdom**	81%	30%	36%	39%	36%	26%	37%
**Bulgaria**	45%	33%	38%	28%	44%	22%	35%
**Romania**	70%	42%	29%	30%	38%	34%	34%
**Slovakia**	-	71%	30%	33%	30%	15%	32%
**Austria**	100%	32%	23%	28%	40%	29%	31%
**Hungary**	100%	75%	36%	35%	24%	16%	31%
**Italy**	65%	53%	39%	30%	26%	15%	29%
**Luxembourg**	-	-	28%	23%	27%	12%	25%
**Croatia**	49%	49%	27%	27%	18%	12%	23%
**Cyprus**	-	-	0%	37%	22%	11%	22%
**Slovenia**	-	0%	12%	19%	21%	13%	17%
**Average**	**69%**	**54%**	**33%**	**35%**	**33%**	**23%**	**34%**

Included causes of death: injuries to the head (S00–S09); injuries involving multiple body regions (T00–T07); injuries to unspecified trunk, limb, or body region (T08–T14); certain early complications of trauma (T79); and sequelae of injuries, of poisoning, and of other consequences of external causes (T90–T98). YLL, year of lost life.
