# Supplementary material for: Years of life lost due to traumatic brain injury in Europe: A cross-sectional analysis of 16 countries
Source: PLoS Med. 2017 Jul 11;14(7):e1002331. doi: 10.1371/journal.pmed.1002331 (PMC5507416; doi:10.1371/journal.pmed.1002331)
Supplement: S13 Table — (PDF) [file pmed.1002331.s016.pdf]

S13 Table: Total numbers of injury YLLs in 16 European countries in 2013 by age-group and sex (all causes of death included)

	Age-group	0 - 4	5 - 14	15 - 34	35 - 64	65 - 84	85+	TOTAL
Total	United Kingdom	6759	6572	180776	254823	57262	11811	518003
	Italy	5888	6602	134746	172307	79807	20260	419610
	Romania	12863	13016	86140	159628	31465	1349	304461
	Hungary	1745	1822	29138	80782	23599	2888	139974
	Lithuania	1026	1398	30962	64396	9244	596	107622
	Austria	1003	1673	25638	41943	18074	3106	91437
	Serbia	1571	1985	25411	41795	12655	617	84034
	Slovakia	944	1884	22467	44151	10240	1197	80883
	Bulgaria	2541	2398	24901	38149	10389	642	79020
	Croatia	858	710	18257	27231	11988	1745	60789
	Ireland	629	896	20075	21937	3656	379	47572
	Denmark	1106	762	10772	22557	6593	1210	43000
	Estonia	1121	571	10880	15870	2807	149	31398
	Slovenia	78	279	8029	14150	5671	876	29083
	Cyprus	0	217	3025	2388	1011	164	6805
	Luxembourg	157	130	1511	3444	1051	162	6455
	**Total**	**38289**	**40915**	**632728**	**1005551**	**285512**	**47151**	**2050146**
	**Proportion of total**	**2%**	**2%**	**31%**	**49%**	**14%**	**2%**	**100%**
Males	United Kingdom	4131	3968	138746	185213	30456	4471	366985
	Italy	3263	4183	107107	132349	45430	6815	299147
	Romania	8021	8140	69307	130141	20503	663	236775
	Hungary	998	1087	22559	61226	13400	869	100139
	Lithuania	617	808	25977	52100	5819	184	85505
	Austria	756	867	19393	31516	11438	1060	65030
	Slovakia	618	1296	18969	35792	6343	372	63390
	Bulgaria	1064	1955	20831	31395	6964	325	62534
	Serbia	915	878	20351	31774	8248	267	62433
	Croatia	530	404	15249	21444	6237	426	44290
	Ireland	304	747	15621	16720	2215	130	35737
	Denmark	692	394	7897	16566	3882	405	29836
	Estonia	462	273	8897	12779	1815	62	24288
	Slovenia	78	203	6392	11179	3254	239	21345
	Cyprus	0	139	2790	1813	638	47	5427
	Luxembourg	77	130	1005	2300	625	49	4186
	**Total**	**22526**	**25472**	**501091**	**774307**	**167267**	**16384**	**1507047**
	**Proportion of total**	**1%**	**2%**	**33%**	**51%**	**11%**	**1%**	**100%**
Females	United Kingdom	2628	2604	42030	69610	26806	7340	151018
	Italy	2625	2419	27639	39958	34377	13445	120463
	Romania	4842	4876	16833	29487	10962	686	67686
	Hungary	747	735	6579	19556	10199	2019	39835
	Austria	247	806	6245	10427	6636	2046	26407
	Lithuania	409	590	4985	12296	3425	412	22117
	Serbia	656	1107	5060	10021	4407	350	21601
	Slovakia	326	588	3498	8359	3897	825	17493
	Croatia	328	306	3008	5787	5751	1319	16499
	Bulgaria	1477	443	4070	6754	3425	317	16486
	Denmark	414	368	2875	5991	2711	805	13164
	Ireland	325	149	4454	5217	1441	249	11835
	Slovenia	0	76	1637	2971	2417	637	7738
	Estonia	659	298	1983	3091	992	87	7110
	Luxembourg	80	0	506	1144	426	113	2269
	Cyprus	0	78	235	575	373	117	1378
	**Total**	**15763**	**15443**	**131637**	**231244**	**118245**	**30767**	**543099**
	**Proportion of total**	**3%**	**3%**	**24%**	**43%**	**22%**	**6%**	**100%**

YLL=Years of Lost Life
