# Supplementary material for: Years of life lost due to traumatic brain injury in Europe: A cross-sectional analysis of 16 countries
Source: PLoS Med. 2017 Jul 11;14(7):e1002331. doi: 10.1371/journal.pmed.1002331 (PMC5507416; doi:10.1371/journal.pmed.1002331)
Supplement: S14 Table — (PDF) [file pmed.1002331.s017.pdf]

S14 Table: Crude and age-adjusted injury YLL rates in 16 European countries in 2013 by age-group and sex (all causes of death included)

	3	0 - 4	5 - 14	15 - 34	35 - 64	65 - 84	85+	crude rate	age-adjusted rate (95% CI)
**Total**	**Lithuania**	679.2	495.3	4037.4	5300.0	1911.3	1013.1	3638.7	3554.2 (3533.0 - 3575.5)
	**Estonia**	1496.6	430.9	3200.9	2990.1	1315.1	562.8	2382.3	2339.1 (2313.2 - 2365.1)
	**Romania**	1309.8	609.6	1683.8	1883.9	1054.6	458.9	1523.5	1490.4 (1485.1 - 1495.7)
	**Slovakia**	324.2	349.5	1433.2	1924.7	1562.1	1809.0	1494.1	1488.9 (1478.5 - 1499.5)
	**Croatia**	405.6	168.8	1706.0	1534.7	1689.0	2560.3	1428.8	1421.0 (1409.7 - 1432.4)
	**Slovenia**	70.4	147.8	1590.7	1573.1	1793.7	2192.5	1411.8	1398.4 (1382.3 - 1414.6)
	**Hungary**	380.0	188.0	1169.1	1898.2	1527.9	1677.2	1414.9	1390.7 (1383.4 - 1398.1)
	**Luxembourg**	511.9	212.3	1037.7	1499.7	1572.4	1727.9	1188.0	1215.9 (1186.0 - 1246.5)
	**Serbia**	474.1	285.0	1424.9	1358.5	1068.6	670.5	1173.0	1145.6 (1137.9 - 1153.5)
	**Bulgaria**	737.7	369.7	1411.5	1230.1	806.9	539.2	1087.7	1065.2 (1057.7 - 1072.6)
	**Austria**	251.9	203.8	1200.3	1170.8	1353.2	1505.3	1078.3	1064.9 (1058.0 - 1071.9)
	**Ireland**	171.0	139.8	1625.2	1230.5	718.1	606.9	1034.6	1049.6 (1040.0 - 1059.3)
	**United Kingdom**	168.4	90.1	1071.6	1026.6	592.6	806.1	807.8	812.2 (810.0 - 814.4)
	**Cyprus**	0.0	237.2	1116.3	716.0	956.9	1480.8	789.5	783.7 (764.5 - 803.3)
	**Denmark**	358.7	114.6	783.8	1000.7	734.1	1050.4	765.8	775.0 (767.7 - 782.4)
	**Italy**	215.6	116.5	1043.4	660.3	725.5	1109.2	696.6	692.5 (690.4 - 694.6)
	**Pooled**								**1355.5 (1083.8 - 1627.1)**
**Males**	**Lithuania**	796.1	558.2	6632.6	9203.4	3442.4	1369.2	6275.8	6039.8 (5998.9 – 6081.0)
	**Estonia**	1202.5	400.8	5083.1	5037.2	2428.1	1133.2	3945.8	3805.3 (3756.9 - 3854.5)
	**Slovakia**	413.8	468.3	2365.2	3153.2	2477.6	2020.6	2403.4	2388.6 (2369.0 - 2408.4)
	**Romania**	1589.0	742.4	2628.6	3100.0	1681.3	673.6	2427.5	2350.2 (2340.6 - 2359.8)
	**Croatia**	486.7	187.0	2792.4	2446.7	2167.8	2349.3	2157.2	2133.3 (2113.0 - 2153.7)
	**Hungary**	423.0	218.4	1771.9	2964.2	2262.9	1914.8	2126.2	2103.5 (2090.2 - 2116.9)
	**Slovenia**	136.3	209.3	2437.4	2432.0	2425.4	2454.2	2092.7	2073.4 (2045.0 - 2102.3)
	**Serbia**	536.0	245.2	2230.9	2106.7	1626.1	838.7	1789.7	1739.8 (1726.1 - 1753.7)
	**Bulgaria**	601.3	586.1	2283.4	2036.0	1314.3	807.3	1769.0	1710.7 (1697.2 - 1724.3)
	**Luxembourg**	488.7	414.3	1359.8	1959.4	2044.2	1804.2	1540.4	1582.5 (1533.4 - 1633.4)
	**Ireland**	162.1	228.1	2559.5	1885.3	911.7	645.0	1570.6	1582.1 (1565.4 – 1599.0)
	**Austria**	369.1	206.2	1791.2	1771.6	1924.6	1822.2	1570.9	1561.3 (1549.2 - 1573.4)
	**Cyprus**	0.0	296.0	2068.2	1143.0	1298.2	1083.7	1294.5	1239.6 (1205.6 - 1274.7)
	**United Kingdom**	201.1	106.2	1632.3	1510.6	676.9	906.3	1163.4	1163.0 (1159.2 - 1166.8)
	**Denmark**	437.5	115.7	1131.5	1465.9	919.5	1097.4	1071.1	1083.6 (1071.2 - 1096.1)
	**Italy**	232.5	143.5	1634.5	1030.8	924.4	1214.0	1024.9	1024.7 (1021.0 - 1028.4)
	**Pooled**								**2098.8 (1585.2 - 2612.5)**
**Females**	**Lithuania**	555.8	429.5	1328.6	1894.8	1088.8	907.5	1386.5	1369.1 (1351.0 - 1387.3)
	**Estonia**	1806.6	462.7	1202.7	1115.5	715.1	413.3	1012.0	1013.5 (990.0 - 1037.5)
	**Luxembourg**	533.7	0.0	705.1	1019.0	1173.6	1708.5	835.1	850.8 (815.9 - 886.9)
	**Hungary**	334.1	155.9	539.6	892.9	1070.9	1592.2	768.5	746.8 (739.5 - 754.2)
	**Croatia**	319.5	149.3	573.9	644.5	1362.6	2637.3	749.5	728.7 (717.6 – 740.0)
	**Slovenia**	0.0	82.5	675.0	675.4	1327.9	2107.9	743.9	723.0 (707.0 - 739.4)
	**Romania**	1014.4	469.5	679.0	689.7	621.4	350.6	661.7	659.1 (654.1 - 664.1)
	**Slovakia**	229.7	224.3	456.9	721.3	975.5	1726.8	630.2	646.9 (637.3 - 656.7)
	**Austria**	127.9	201.2	593.0	578.2	895.0	1380.8	608.5	591.7 (584.5 - 598.9)
	**Serbia**	408.1	327.2	580.8	639.0	651.0	581.3	587.7	579.8 (572.0 - 587.6)
	**Ireland**	180.5	47.7	712.8	582.3	541.6	588.5	509.5	528.0 (518.3 - 537.8)
	**United Kingdom**	134.2	73.1	502.2	554.2	519.2	755.2	463.5	466.9 (464.5 - 469.2)
	**Denmark**	275.8	113.5	425.1	533.0	569.6	1028.6	465.3	466.7 (458.7 - 474.7)
	**Bulgaria**	881.1	140.6	477.8	433.2	452.0	403.0	442.0	436.6 (429.9 - 443.3)
	**Italy**	197.8	87.9	434.5	301.5	564.8	1062.7	388.0	368.5 (366.4 - 370.6)
	**Cyprus**	0.0	174.6	172.9	328.6	660.5	1743.0	311.2	349.6 (331.0 - 369.2)
	**Pooled**								**657.9 (549.0 - 766.7)**

Meta-analysis heterogeneity: I^2 for total= 100% (95%CI: 100% to 100%); I^2 for males= 100% (95%CI: 100% to 100%); I^2 for females= 99.9% (95%CI: 99.9% to 99.9%);

YLL=Years of Lost Life
