# Supplementary material for: Years of life lost due to traumatic brain injury in Europe: A cross-sectional analysis of 16 countries
Source: PLoS Med. 2017 Jul 11;14(7):e1002331. doi: 10.1371/journal.pmed.1002331 (PMC5507416; doi:10.1371/journal.pmed.1002331)
Supplement: S15 Table — (PDF) [file pmed.1002331.s018.pdf]

S15 Table: Contribution of TBI YLLs to injury YLLs in 16 European countries in 2013 by age-group and sex (all causes of death included)

Age-group	0 - 4	5 - 14	15 - 34	35 - 64	65 - 84	85+	TOTAL
Cyprus	-	0	40%	24%	22%	11%	30%
Serbia	20%	54%	31%	23%	29%	21%	27%
Bulgaria	34%	26%	23%	22%	25%	19%	23%
Estonia	29%	37%	19%	22%	27%	20%	22%
Italy	30%	30%	24%	19%	25%	16%	22%
Slovakia	8%	38%	19%	20%	30%	19%	21%
Austria	31%	28%	16%	19%	30%	28%	21%
Croatia	36%	39%	20%	21%	20%	15%	21%
Luxembourg	0	50%	28%	17%	19%	17%	20%
Denmark	22%	65%	26%	15%	21%	12%	20%
Romania	19%	18%	18%	17%	23%	25%	18%
Hungary	27%	20%	21%	15%	20%	17%	18%
Ireland	25%	55%	14%	12%	25%	30%	15%
Lithuania	8%	21%	12%	14%	22%	20%	14%
United Kingdom	30%	19%	11%	11%	26%	27%	13%
Slovenia	0	0	10%	11%	16%	15%	12%
**Average**	**21%**	**31%**	**21%**	**18%**	**24%**	**20%**	**20%**
Cyprus	-	0%	44%	24%	23%	16%	33%
Serbia	25%	46%	32%	25%	31%	27%	28%
Luxembourg	0%	50%	31%	21%	23%	31%	25%
Bulgaria	50%	28%	22%	23%	26%	26%	24%
Estonia	17%	24%	22%	24%	31%	23%	24%
Italy	21%	32%	24%	20%	28%	20%	23%
Croatia	43%	32%	20%	23%	25%	27%	22%
Austria	20%	39%	17%	20%	33%	37%	22%
Slovakia	13%	27%	18%	22%	33%	31%	22%
Denmark	11%	50%	25%	17%	24%	16%	20%
Romania	16%	14%	18%	18%	25%	33%	18%
Hungary	31%	12%	22%	16%	24%	24%	18%
Ireland	25%	55%	16%	13%	22%	29%	16%
Lithuania	0%	17%	13%	14%	26%	21%	14%
United Kingdom	24%	24%	11%	11%	28%	32%	13%
Slovenia	0%	0%	11%	11%	19%	25%	12%
**Average**	**20%**	**28%**	**21%**	**19%**	**26%**	**26%**	**21%**
Serbia	13%	60%	30%	18%	25%	16%	24%
Bulgaria	22%	17%	25%	17%	23%	13%	20%
Italy	41%	27%	23%	18%	22%	14%	20%
Slovakia	0%	63%	21%	15%	24%	14%	19%
Denmark	40%	81%	28%	11%	17%	11%	19%
Austria	66%	17%	14%	13%	25%	24%	18%
Romania	24%	24%	17%	13%	20%	17%	17%
Estonia	37%	50%	7%	14%	19%	18%	16%
Croatia	25%	49%	19%	14%	14%	12%	15%
Hungary	22%	31%	19%	13%	16%	13%	15%
Cyprus	-	0%	0%	22%	20%	10%	15%
Ireland	25%	50%	11%	10%	31%	30%	14%
United Kingdom	41%	12%	12%	10%	24%	24%	14%
Lithuania	20%	26%	9%	13%	16%	20%	13%
Luxembourg	0%	-	22%	8%	14%	10%	12%
Slovenia	-	0%	7%	8%	12%	12%	9%
**Average**	**27%**	**34%**	**17%**	**14%**	**20%**	**16%**	**16%**

YLL=Years of Lost Life, TBI=Traumatic Brain Injury
