# Supplementary material for: Years of life lost due to traumatic brain injury in Europe: A cross-sectional analysis of 16 countries
Source: PLoS Med. 2017 Jul 11;14(7):e1002331. doi: 10.1371/journal.pmed.1002331 (PMC5507416; doi:10.1371/journal.pmed.1002331)
Supplement: S1 Text — (PDF) [file pmed.1002331.s019.pdf]

Years of lost life due to traumatic brain injury in Europe

Original analysis plan of the study (conceived November 14th, 2016)

1) Objectives and hypotheses

Traumatic brain injuries (TBI) are a major public health and societal challenge globally. In order to describe the true population burden of TBI, epidemiological reports must provide in-depth analyses which go beyond standard epidemiological indicators (such as mortality or incidence) and estimate the impact these injuries have on the individual and societal level. A recent analysis estimated that TBI result in about 1.5 million hospital admissions and about 57,000 deaths annually in the European Union (EU) only. Based on these analyses, when planning and designing our study we hypothesized that throughout Europe TBI is associated with a substantial number of years of lost life – mainly due to the fact that a large proportion of TBI related fatalities occur in younger age-groups.

2) Analytical methods planned

To test this hypothesis we intended to acquire recent and detailed data on TBI related deaths from European countries. We aimed to contact Eurostat as the statistical authority of the EU which oversees the collection of data on causes of death and regulates the practices of coding across the countries in order to make these data comparable across the EU. Our goal was to acquire the most recent data that was available for as many European countries as possible in a unified and comparable format. In order to calculate the number of YLLs as precisely as possible we intended to apply for microdata on injury related deaths where for each case the year of death, exact age at death (number of years of age at death), sex, country, ICD10 code for the external cause of injury and ICD10 code for the nature of injury leading to death would be recorded. In the next step we planned to select a subset of deaths that were caused by TBI – based on ICD10 codes for the nature on injury – ICD10 codes S00-S09 (injuries to the head) and T90 (sequalae of injuries to the head) were considered as indicators of TBI deaths.

We planned to calculate the following variables and present them stratified by country, sex, and age-group:

a) Number of TBI YLLs
b) Crude rate of TBI YLLs per 100,000 persons
c) Crude rate of TBI YLLs per death case;

In order to control for the differences in the age-structure of the various EU countries and in order to present results that would be comparable across the analyzed countries we have planned to present age-standardized rates of TBI YLLs per 100,000 and TBI YLLs per death case for each country and sex with 95% confidence intervals. We intended to apply direct age-standardization using the 2013 European standard population.

To present a summary measure for EU, we planned to calculate pooled age-standardized rates based on the age-standardized rates calculated for each country. As we expected a large degree of heterogeneity, we planned to apply the random-effects model by the DerSimonian and Laird method to calculate pooled estimates. To assess heterogeneity of the pooled estimations, we aimed to calculate and present I^2 with 95% confidence intervals.

3) Analyses that were actually performed

We have successfully obtained the microdata in the format that we have planned from Eurostat for 16 European countries for the year 2013. This allowed us to follow our original analysis plan – all analyses that were planned were performed during the study.

4) Ethical consideration

As the study only planned to use routinely collected secondary data, no ethics approval was required, and thus this study plan was not part of an IRB proposal or other application.
